# Nutritional properties of extracellular vesicle-like particles from *Sophora flavescens* and *Periplaneta americana* with effects on streptozotocin-induced diabetic wound healing in rats

**DOI:** 10.3389/fnut.2026.1807919

**Published:** 2026-04-29

**Authors:** Zhengting Wu, Yuanxin Zhao, Weiyin Zeng, Zesheng Lu, Qi You, Yingqi Cao, Yuanyuan Xia, Wencong Shen, Zilin Ou, Xiuping Cai, Qing Zhao, Kewei Zhao

**Affiliations:** 1 The Third Clinical Medical College, Guangzhou University of Chinese Medicine, Guangzhou, China; 2 State Key Laboratory of Traditional Chinese Medicine Syndrome, The Third Affiliated Hospital of Guangzhou University of Chinese Medicine, Guangzhou, China; 3 Guangdong Engineering Research Center of Chinese herbal-derived vesicles, Guangzhou University of Chinese Medicine, Guangzhou, China; 4 Guangdong Pilot Platform for Chinese Herbal Medicine-derived Extracellular Vesicles-like Particles, The Third Affiliated Hospital of Guangzhou University of Chinese Medicine, Guangzhou, China

**Keywords:** cross-kingdom modulation, diabetic wound, EGFR signaling pathway, *Periplaneta americana*-derived extracellular vesicle-like particles, *Sophora flavescens*-derived extracellular vesicle-like particles

## Abstract

**Introduction:**

The medicinal plant *Sophora flavescens* (SF) and the insect *Periplaneta americana* (PA) have long been used in treating diabetic wounds. PA-derived extracellular vesicle-like particles (PA-EVLP) can accelerate diabetic wound healing. However, no evidence of the nutritional components and anti-diabetic wound effects of the combination of SF-EVLP with PA-EVLP (SF-PA-EVLPs) is available.

**Methods:**

We separated SF-EVLP and PA-EVLP via ultracentrifugation and characterized them via nano flow cytometry, transmission electron microscopy, and atomic force microscopy. Ultra-high-performance liquid chromatography coupled with mass spectrometry (UPLC/MS) was used to analyze the nutritional content of proteins, lipids, amino acids, phenols, and flavonoids in SF-EVLP and PA-EVLP. Moreover, we comprehensively evaluated the effects of SF-PA-EVLPs on wound healing and related complications in diabetic rats and systematically elucidated the underlying mechanisms.

**Results:**

The size of SF-EVLPs and PA-EVLPs was in the nanometer range, and they had a lipid bilayer structure. They contained abundant bioactive substances, lipids, proteins, and nucleic acids. In diabetic rat models, SF-PA-EVLPs had better wound healing effects than PA extract alone and significantly promoted diabetic wound healing. Concurrently, SF-PA-EVLPs had negligible effects on bone formation in streptozotocin-induced diabetic rats while ameliorating pancreatic injury. Moreover, several key substances within SF-PA-EVLPs exhibited significant enrichment in the EGFR signaling pathway with strong binding affinity.

**Conclusion:**

At the dosage and treatment duration used in this study, combined therapy with SF-PA-EVLPs had stronger diabetic wound healing effects compared to clinically used PA extracts. Here, we also established the first cross-kingdom modulation system, providing a novel approach for developing nano-nutritional therapeutics based on medicinal plants, edible insects, and foods.

## 1 Introduction

Diabetes is a severe global health challenge. The International Diabetes Federation estimates that 537 million adults were living with the disease in 2021, and they predict that this number will increase to 783 million by 2045 (1). Diabetic wounds, among the most severe complications of diabetes, have high incidence rates and high disability rates and impose significant economic burdens. The annual incidence of diabetic foot ulcers in the global diabetic population is approximately 2%, with a lifetime risk of 25% (2, 3). However, no evidence of nutritional therapeutics with high efficacy and low toxicity for diabetic wound healing is available.

Diabetic wounds are treated using various medicinal plants and insects. For example, *Sophora flavescens* (SF) has been used for thousands of years in China to treat diabetic wounds. SF contains several chemical components, including matrine and oxymatrine, which exhibit anti-inflammatory and antimicrobial activities (4, 5). Moreover, in China, PA is processed into the commercial medicine “Kangfuxin Liquid” (6, 7). PA-derived extracellular vesicle-like particles (PA-EVLP) accelerate diabetic wound healing (8), and PA has been used for centuries (9).

Extracellular vesicle-like particles (EVLP) have emerged as promising therapeutic agents. EVLP can effectively encapsulate diverse bioactive components from their source materials, including lipids, proteins, and nucleic acids. Their nanoscale phospholipid bilayer structure increases cellular uptake, stability, and bioavailability (10, 11). However, no study has addressed the nutritional components and combined anti-diabetic wound effects of SF-EVLP with PA-EVLP on streptozotocin (STZ)-induced diabetic wounds in rats or their associated mechanisms. Therefore, in this study, we prepared SF-EVLP and PA-EVLP via differential centrifugation and analyzed the components present in the combination of SF-EVLP with PA-EVLP (SF-PA-EVLPs) using LC-MS. We also analyzed the nutritional properties, including lipids, proteins, nucleic acids, and bioactive substances. Finally, we evaluated the improvement effect and preliminarily explored the mechanism of action of SF-PA-EVLPs in a constructed rat model of STZ-induced diabetic wounds.

## 2 Results

### 2.1 Physical characterization of SF-EVLP and PA-EVLP

**Figure 1:**
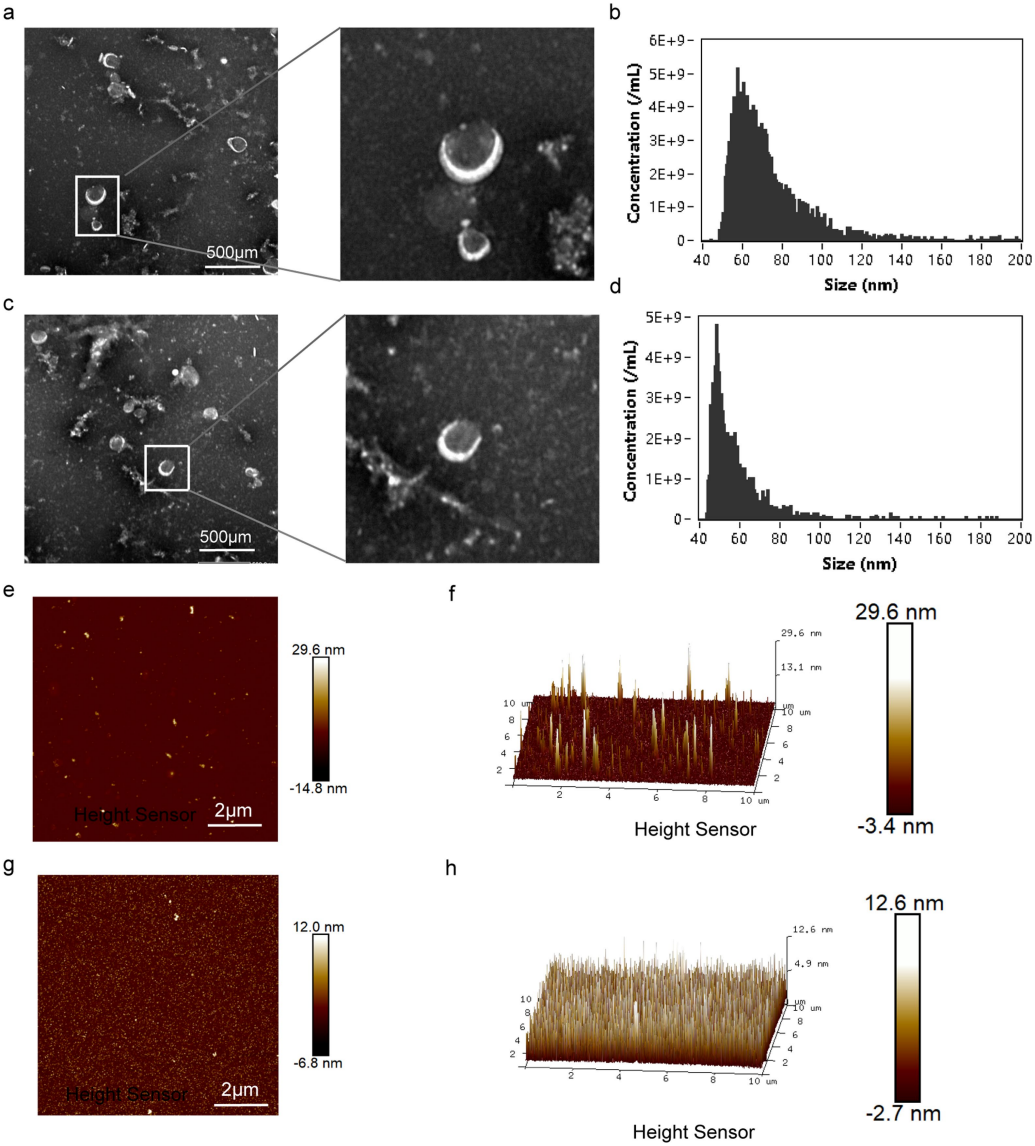
Characterization of SF-EVLP and PA-EVLP. **(a)** Transmission electron microscopy (TEM) images of SF-EVLP are shown; scale bar = 500 nm. **(b)** Size distribution profiles of SF-EVLP are illustrated. **(c)** TEM images of PA-EVLP are shown; scale bar = 500 nm. **(d)** Size distribution profiles of PA-EVLP are illustrated. **(e)** Atomic force microscopy (AFM) topographical analysis of SF-EVLP was performed. **(f)** Three-dimensional AFM visualization of the surface of SF-EVLP. **(g)** AFM topographical analysis of PA-EVLP was performed. **(h)** Three-dimensional AFM visualization of the surface of PA-EVLP.

*Sophora flavescens* (SF) is a medicinal plant commonly used to treat bacterial infections. The small diameter (in the nanometer range) and lipid-bilayer architecture of PD-EVLP provide a large surface area that increases the solubility, stability, and bioavailability of poorly soluble compounds (12). The bilayer structure further allows sustained release and targeted delivery and decreases toxicity (4, 13, 14). We first isolated SF-derived extracellular vesicle-like particles (SF-EVLP) by differential centrifugation and then characterized them for composition and structure. TEM images revealed that SF-EVLPs were uniform in size and either round or cup-shaped (Figure 1a). Nano-flow analysis revealed that they were less than 200 nm (Figure 1b). *Periplaneta americana* (PA) is an animal commonly used in wound healing. TEM images showed that PA-EVLPs were uniform in size and either round or cup-shaped (Figure 1c). Nano-flow analysis showed that they were less than 200 nm (Figure 1d). Atomic force microscopy (AFM) was used to observe the surface morphology of SF-EVLP and PA-EVLP and revealed white spots distributed across their surfaces. This indicated that SF-EVLP (Figures 1e,f) and PA-EVLP had a uniformly spherical shape (Figures 1g,h). High-resolution 3D AFM imaging confirmed the spherical vesicle-like structure of SF-PA-EVLPs.

### 2.2 Purity of SF-EVLP and PA-EVLP

Based on membrane structure, we performed membrane solubilization using Triton X-100, which disrupts the membrane’s integrity, thereby allowing purification of EVLP to be assessed (15). Compared with an absence of Triton X-100, the number of particles in SF-EVLP when treated with 0.1% Triton X-100 decreased significantly to 12.2%. And the number of particles in PA-EVLP when treated with 0.1% Triton X-100 decreased significantly to 39.1%. It may be attributed to the fact that the membrane structure of PD-EVLPs is denser, more rigid, and more stable due to its enrichment in saturated fatty acids and phytosterols, thereby exhibiting stronger resistance to membrane-disrupting agents such as Triton X-100. In contrast, the membrane structure of insect-derived EVLPs, which requires high fluidity, is enriched in unsaturated fatty acids and cholesterol, rendering it relatively loose and fragile. Consequently, it is more susceptible to disruption by membrane-disrupting agents, leading to a more pronounced decrease in particle count.

### 2.3 Nutritional components of SF-EVLP and PA-EVLP

**Figure 2:**
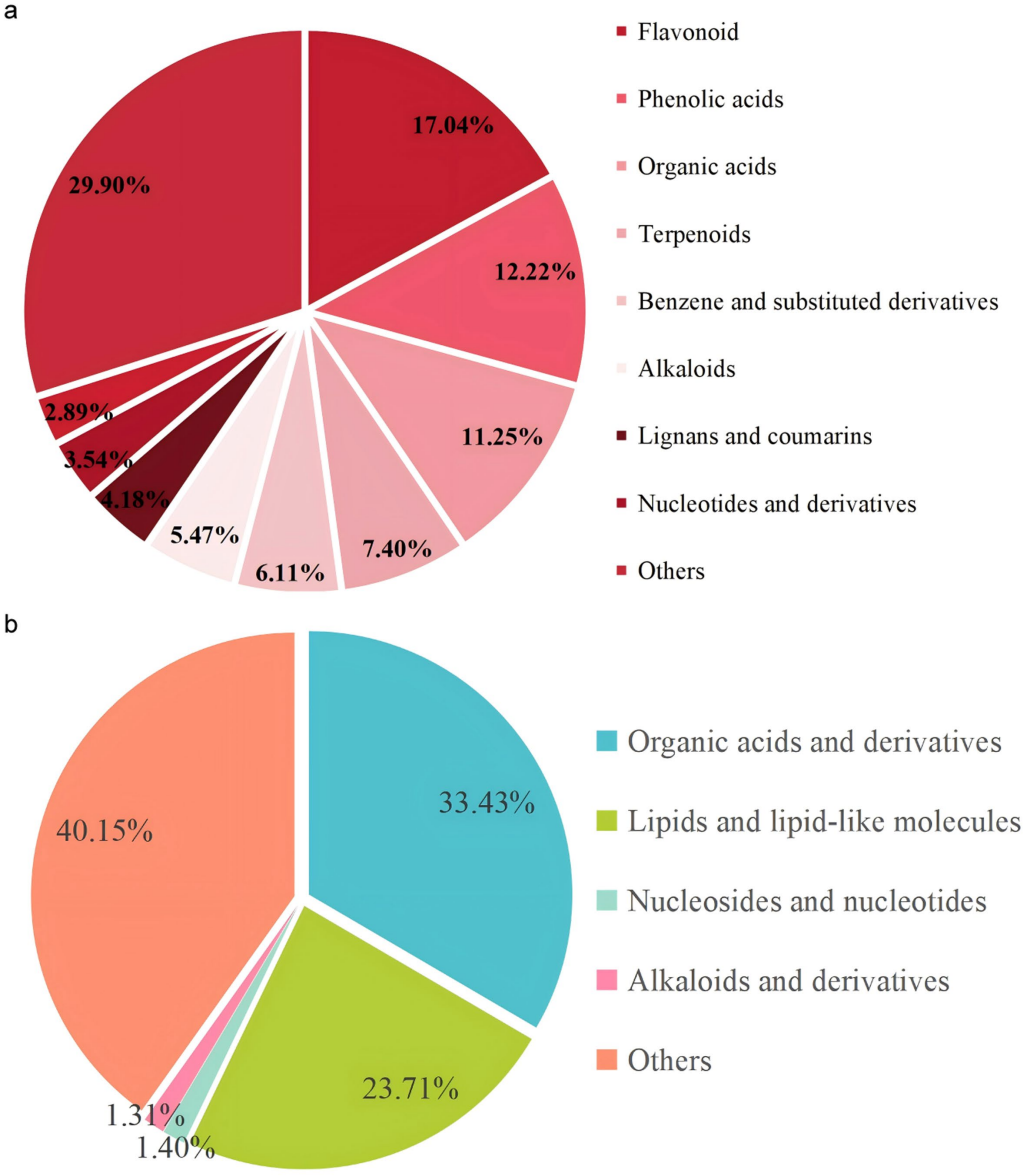
Characterization and identification of the components in SF-EVLP and PA-EVLP. Classification of compounds contained in **(a)** SF-SF-EVLP and **(b)** PA-EVLP.

Exosomes contain various nutrients derived from their parent plants. The most distinctive feature of exosomes is their phospholipid bilayer structure. By conducting liquid chromatography-mass spectrometry (LC-MS) analysis, we identified 15 classes of phytochemicals in SF-EVLP, including flavonoids, phenolic acids, terpenoids, alkaloids, lignans, and coumarins (Figure 2a). In PA-EVLP, we identified 17 components, including lipids and lipid-like molecules, organic acids and derivatives, alkaloids and derivatives, nucleosides, nucleotides, and analogs, among others (Figure 2b). Thus, SF-EVLP and PA-EVLP have a rich diversity of constituents.

### 2.4 The chemical fingerprint of SF-EVLP and PA-EVLP

**Figure 3:**
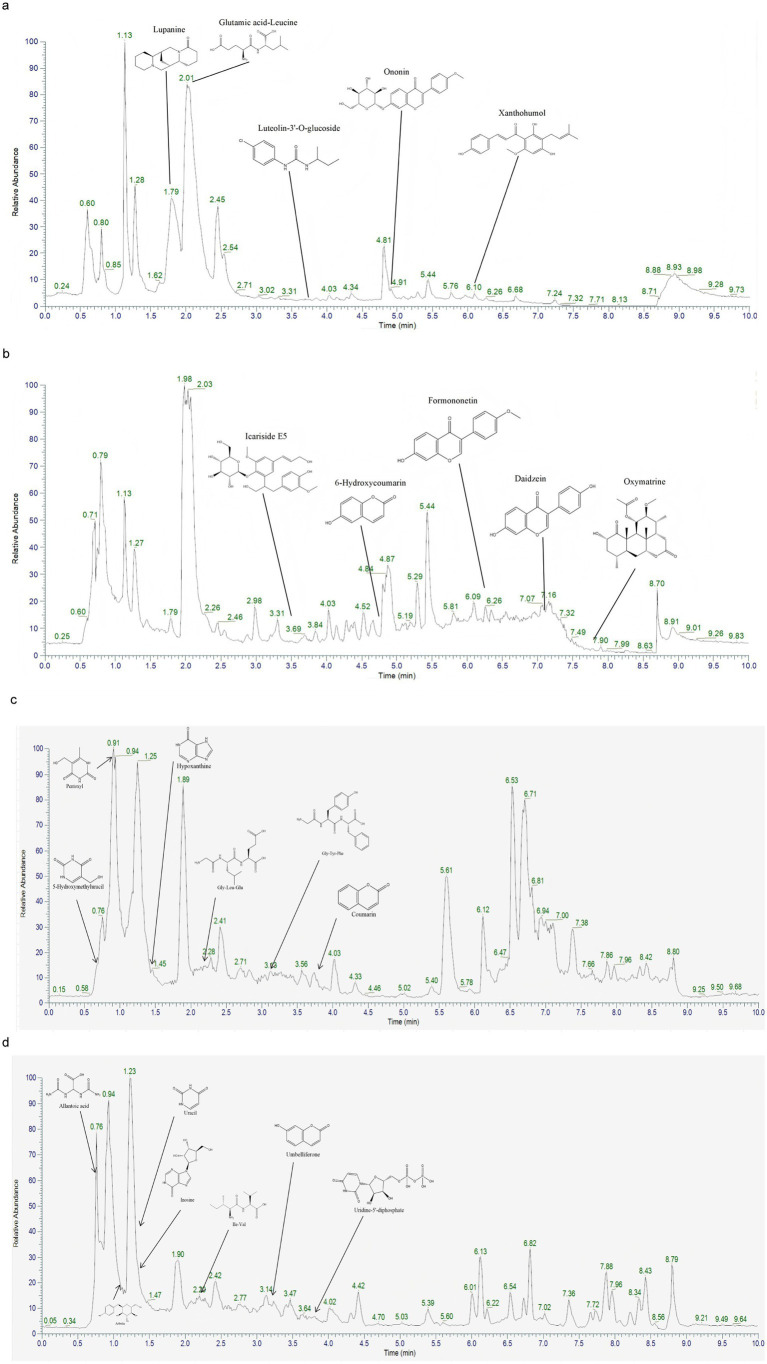
The chemical fingerprint of SF-EVLP and PA-EVLP was constructed by UPLC-MS analysis. **(a)** The chemical fingerprint of SF-EVLP in the positive mode is illustrated. **(b)** The chemical fingerprint of SF-EVLP in the negative mode is illustrated. **(c)** The chemical fingerprint of PA-EVLP in the positive mode is illustrated. **(d)** The chemical fingerprint of PA-EVLP in the negative mode is illustrated.

To reveal the complex chemical profile of SF-EVLP and PA-EVLP, we performed UPLC-MS analysis and established their chemical fingerprint. Using this approach, we identified characteristic compounds, including oxymatrine, formononetin, arbutin, daidzein, and allantoic acid (Figures 3a–d). The in-depth molecular profiling of SF-EVLP and PA-EVLP can improve our understanding of their molecular framework and functions in biological systems.

### 2.5 SF-PA-EVLPs accelerates diabetic skin wound healing

**Figure 4:**
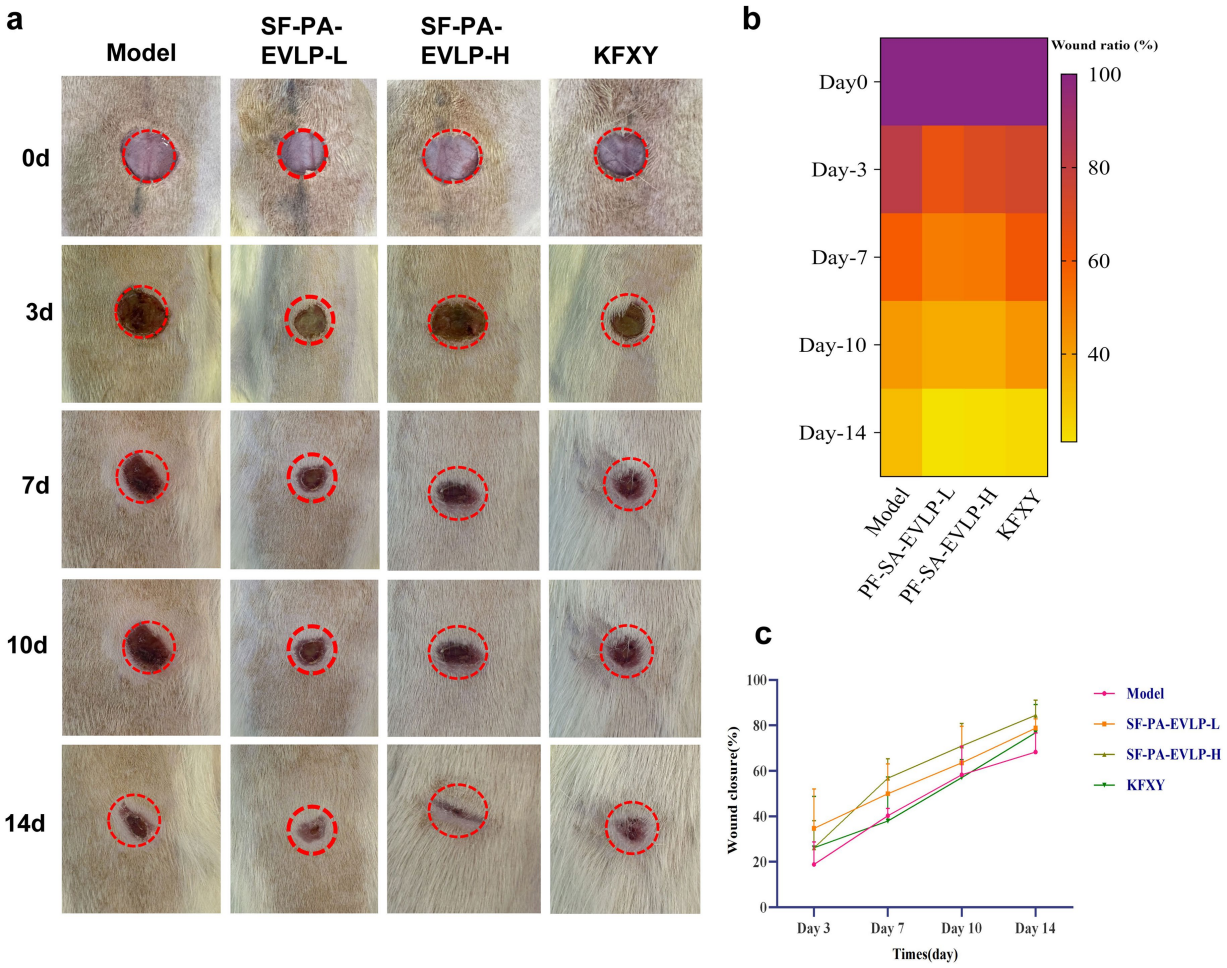
SF-PA-EVLP accelerated diabetic full-thickness back skin wound healing in rats. **(a)** Representative photographs of the back wound healing process after different treatments are presented. **(b,c)** Quantitative analysis of the wound ratio and wound closure rate was performed.

As SF-EVLP has antibacterial properties and PA-EVLP has an excellent wound-healing capacity, we expected SF-PA-EVLPs to have a promising diabetic wound healing effect. We established a streptozotocin (STZ)-induced diabetic rat full-thickness back skin defect model (10 mm in diameter) to assess the healing-acceleration performance of SF-PA-EVLPs (Figure 4a). SF-PA-EVLP (low concentration), SF-PA-EVLP (high concentration), or KFXY were applied once to treat excisional wounds, and untreated wounds served as the model. The wounds treated with SF-PA-EVLP (high concentration) (Figure 4b) exhibited a remarkably accelerated wound closure rate (close to 90.00%) compared to those in the other groups after 14 days of treatment (Figure 4c).

### 2.6 SF-PA-EVLPs enhances collagen formation in diabetic wounds

**Figure 5:**
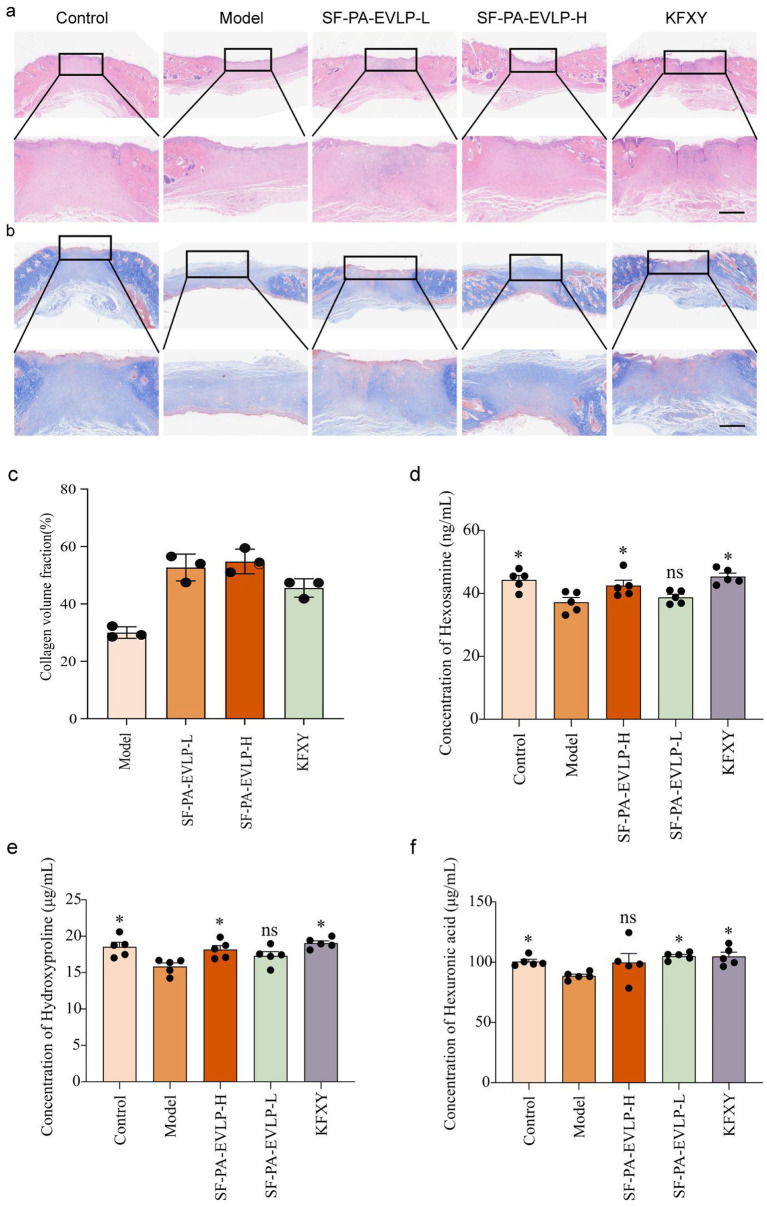
SF-PA-EVLPs accelerated diabetic full-thickness back skin wound healing in rats. **(a)** H&E staining of back wound tissues on day 14; scale bar: 400 μm. **(b,c)** Masson staining and collagen volume fraction of back wound tissues on day 14; scale bar: 400 μm. **(d–f)** Changes in the expression levels of hydroxyproline, hexosamine, and hexuronic acid in serum. The data are presented as the mean ± SD; statistical differences were determined by conducting one-way ANOVA with Tukey’s *post-hoc* test. Compared to model group, *0.01 < *p* < 0.05 and ***0.0001 < *p* < 0.001.

To evaluate the therapeutic effects of different treatments, regenerated wound tissue samples from the four groups were collected on day 14 for histological analysis, including hematoxylin and eosin (H&E) staining and Masson staining (Figures 5a,b). During histopathological evaluation, we focused on wound healing parameters, including the collagen fraction (16). Compared to the model group and the KFXY group, the SF-PA-EVLP group presented more densely packed collagen fibers with well-organized alignment, suggesting high collagen deposition in this group (Figure 5c). Notably, studies have shown that the expression of hydroxyproline, hexosamine and hexuronic acid are positively correlated with collagen content and is closely associated with wound healing (17). Furthermore, measuring serum levels of hydroxyproline, hexosamine, and hexuronic acid is of great practical significance for assessing collagen metabolism and serves as an effective reference indicator for evaluating tissue repair. Among these, hydroxyproline is particularly representative and has been widely used as a non-invasive biomarker for the balance between collagen synthesis and degradation (18, 19). Therefore, we utilized ELISA (competitive method) to measure the expressions of hydroxyproline, hexosamine and hexuronic acid in the serum of rats from each group. Results revealed that compared to the model group, the significant increase in hydroxyproline, hexosamine and hexuronic acid levels following SF-PA-EVLPs treatment directly indicated an increase in collagen content (Figures 5d–f). Consequently, SF-PA-EVLPs treatment accelerates diabetic wound healing.

### 2.7 SF-PA-EVLPs can regulate blood glucose levels in diabetic rats

**Figure 6:**
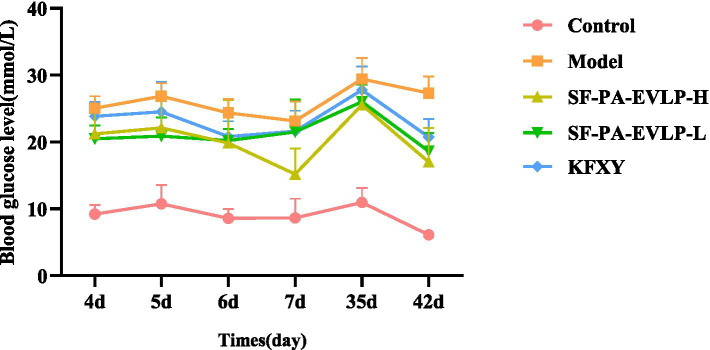
Changes in blood glucose levels in rats after STZ injection.

Blood glucose measurement is particularly important for maintaining the stability of the diabetic model and evaluating diabetic wound treatment. Therefore, after STZ injection, blood glucose was measured at various time points, including days 3, 4, 5, 6, 35 (7 days after SF-PA-EVLPs treatment), and 42 (14 days after SF-PA-EVLPs treatment). The results showed that throughout the experimental observation period, fasting blood glucose levels in both the model control group and the various treatment groups remained consistently above 16.7 mmol/L (Figure 6), indicating that the diabetic model was successfully induced and remained stable. Furthermore, compared with the model group, blood glucose levels in the treatment groups showed a decreasing trend, suggesting that SF-PA-EVLPs exerts a regulatory effect on blood glucose levels in diabetic rats.

### 2.8 SF-PA-EVLPs have negligible effects on bone formation in diabetic wound model rats

The development of diabetogenic osteoporosis is closely associated with a chronic hyperglycemic microenvironment (20). To determine the development of secondary osteoporosis in STZ-induced diabetic rats without high-fat feeding during the experimental period, we conducted histological analysis via femur Masson staining (Supplementary Figure S1a). Under the experimental conditions and dosing regimen used in this study, no significant differences in new bone formation were observed in the distal femurs of STZ-induced diabetic rats compared to those of the rats in the control group (Supplementary Figure S1b). This confirmed that diabetic osteoporosis requires long-term diabetic models for detection.

### 2.9 Observed pancreatic function changes with SF-PA-EVLP treatment

Histopathological observations of pancreatic tissue via H&E staining revealed that in the control group, pancreatic islets exhibited well-rounded morphology, large volume, and normal structure (Supplementary Figure S2). In contrast, in diabetic rats, the pancreatic islets were damaged and degenerated, and the pancreatic islet cells showed marked nuclear atrophy, reduction, and vacuolation. These findings indicate that STZ-induced diabetic rats present islet injury and may mimic certain features of type 2 diabetes. Together with dynamic blood glucose changes, islet injury assessment lends support to the establishment of the diabetic model.

### 2.10 Biosafety evaluation of SF-PA-EVLPs *in vivo*

**Figure 7:**
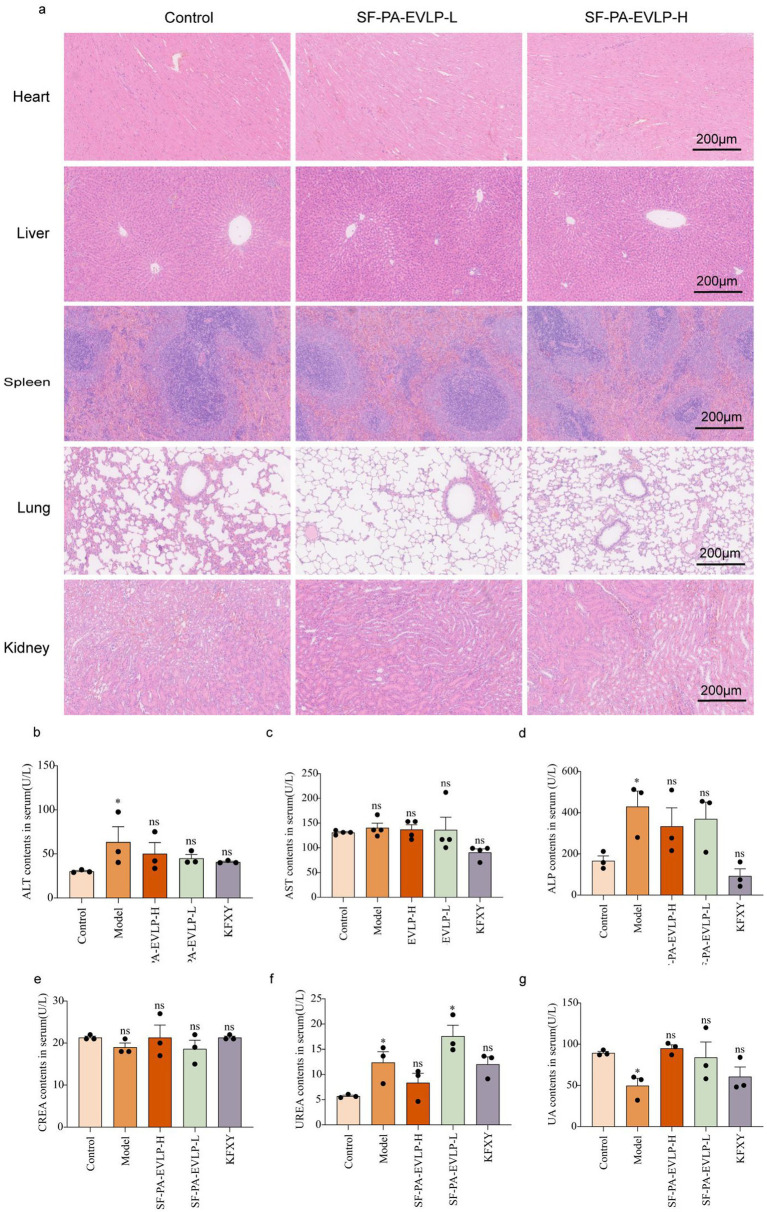
*In vivo* biosafety of SF-PA-EVLPs. **(a)** Histopathological examination via H&E staining of major organs (heart, liver, spleen, lung, and kidney). **(b–g)** Blood biochemical parameters including ALT, AST, ALP, CREA, UREA, and UA were measured to evaluate the systemic safety of SF-PA-EVLP.

Several studies have shown that animal exosomes and plant-derived EVLPs are inert and stable (10, 21). However, the biocompatibility of SF-PA-EVLPs has not been investigated. SF-PA-EVLPs were confirmed to be safe by biosafety testing, which was the first step in assessing the biological applications of SF-PA-EVLPs. H&E staining of the heart, liver, spleen, lungs, and kidneys was conducted following the administration of SF-PA-EVLPs. Histological analyses revealed that no significant damage occurred in any group of rats, which indicated that the material had a good biosafety profile (Figure 7a). These findings obtained from *in vivo* assessments revealed that intra-articular injections of SF-PA-EVLPs have excellent safety profiles, indicating that they are viable therapeutic agents with negligible adverse effects. Furthermore, to evaluate the biosafety of SF-PA-EVLPs. We tested the serum levels of AST, ALT, ALP, urea, UA and CREA showed all the above indicators were within the normal range, indicating that SF-PA-EVLPs did not significantly harm vital organs (Figures 7b–g).

### 2.11 Target collection, PPI network construction, and analysis for SF-PA-EVLPs action against diabetic wounds

**Figure 8:**
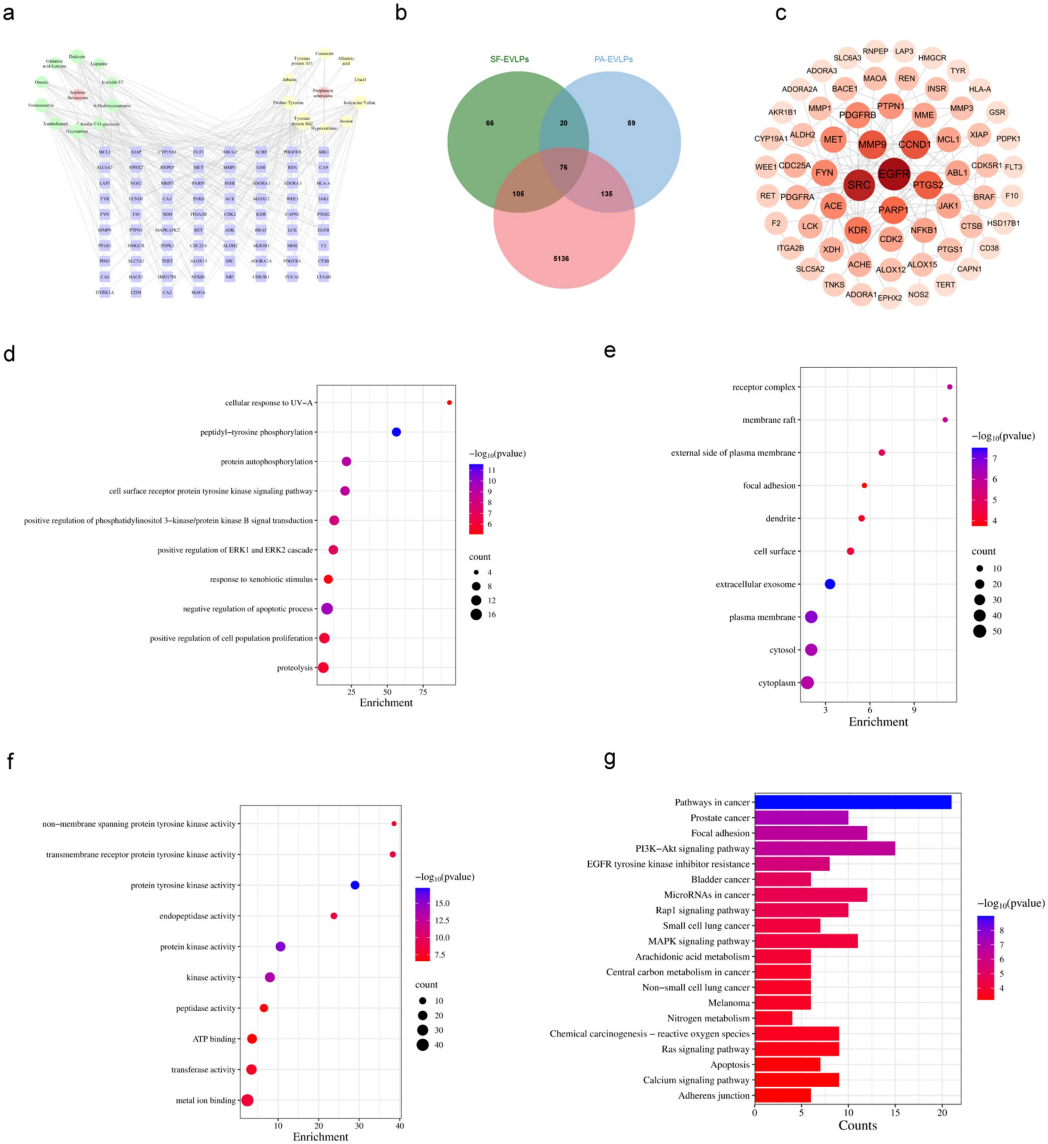
The network of SF-PA-EVLPs-disease intersection targets. **(a)** The component-target network. **(b)** Venn diagram. **(C)** The PPI network. **(d–f)** Histogram of GO enrichment analysis. **(g)** Histogram of KEGG enrichment analysis.

To investigate the mechanism underlying the action of SF-PA-EVLPs in diabetic wound healing, we first investigated the therapeutic effects of SF-PA-EVLPs on diabetic wound healing by network pharmacology. The targets of SF-EVLP and PA-EVLP were predicted using the Swiss Target Prediction platform. The data were systematically summarized and deduplicated, which resulted in 267 and 290 targets (Figure 8a). A database search for targets associated with diabetic ulcers (DU) predicted and screened 76 potential therapeutic targets (Figure 8b). These 76 potential targets were imported into the STRING database to construct a protein interaction network (Supplementary Figure S3), which was visualized and reconstructed using the Cytoscape software. Node size in the network corresponded to degree values, reflecting the importance of the targets. The top 10 core nodes (EGFR, SRC, MMP9, CCND1, PTGS2, and PARP1) were identified as primary therapeutic targets (Figure 8c). GO enrichment analysis and KEGG pathway analysis were performed using the DAVID platform. The top 10 terms for biological process (BP), cellular component (CC), and molecular function (MF) are presented in Figures 8d–f. Additionally, detailed information on the top 20 signaling pathways is provided in the KEGG analysis. The key pathways identified include cancer pathways, the PI3K-Akt signaling pathway, and the Ras signaling pathway (Figure 8g).

### 2.12 Preliminary validation of key components and targets of SF-PA-EVLPs for the treatment of diabetic wounds

**Figure 9:**
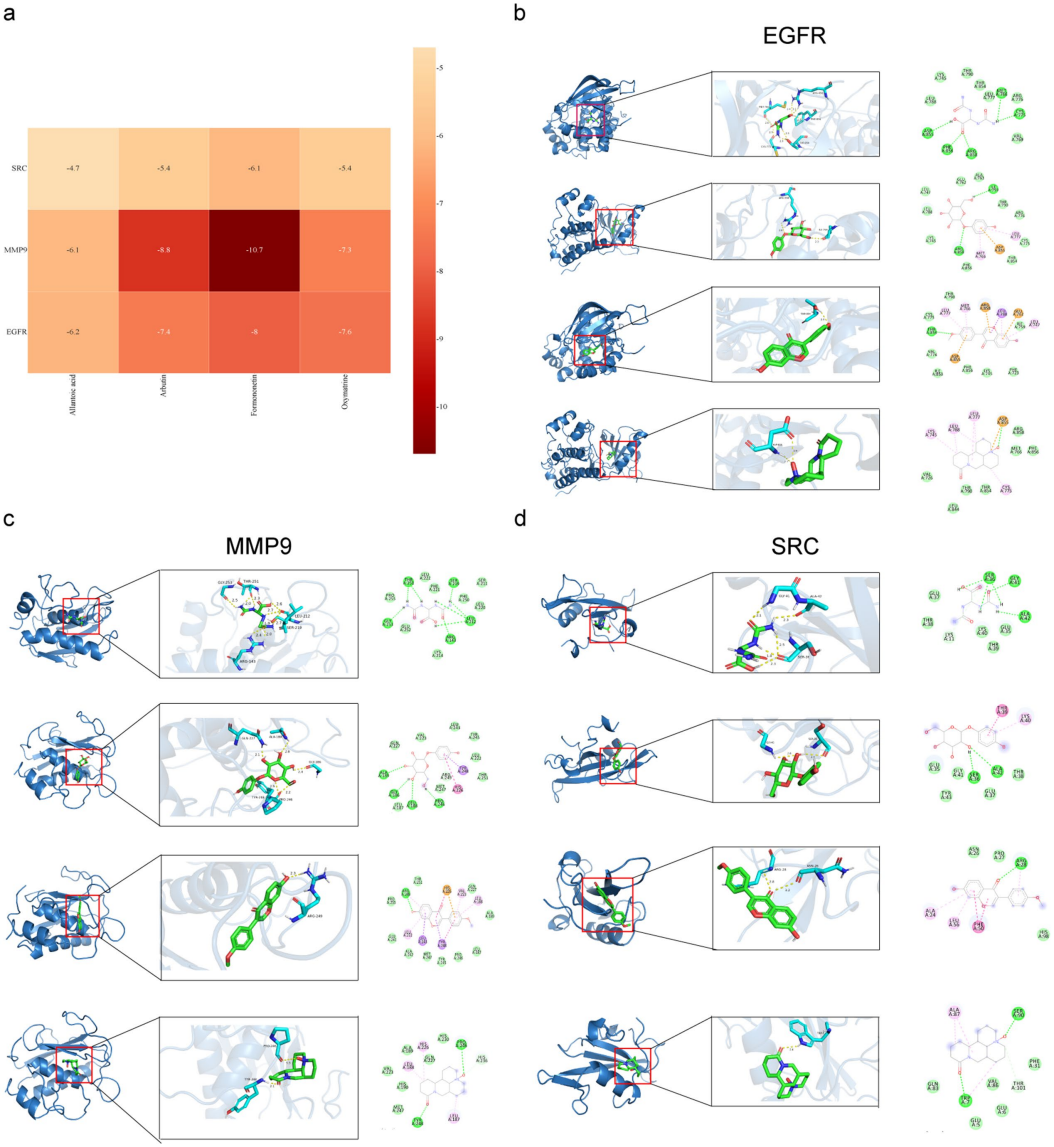
Visualization of molecular docking. **(a)** Affinity ranking of SF-PA-EVLPs binding to DU targets. **(b)** EGFR, **(c)** MMP9, **(d)** SRC, and (1) allantoic acid, (2) arbutin, (3) oxymatrine, and (4) formononetin; for example, a1 is the molecular docking of EGFR and allantoic acid.

The results of the LC-MS analysis of SF-EVLP and PA-EVLP showed that the key ingredients were arbutin, allantoic acid, oxymatrine, and formononetin. The essential targets were EGFR, MMP9, and SRC. They were studied by conducting molecular docking simulations using Auto Dock Tools, and 12 docking results were obtained (Figure 9a). A lower binding energy suggested that the ingredient has a stronger affinity for the target. All groups had a binding energy below −5, upsite allantoic acid and SRC, indicating that binding between the ingredients can occur spontaneously. The 16 docking modes were visualized using the PYMOL software and are shown in Figures 9b–d.

### 2.13 Stable multi-target conjugation of multiple components (SF-PA-EVLPs)

**Figure 10:**
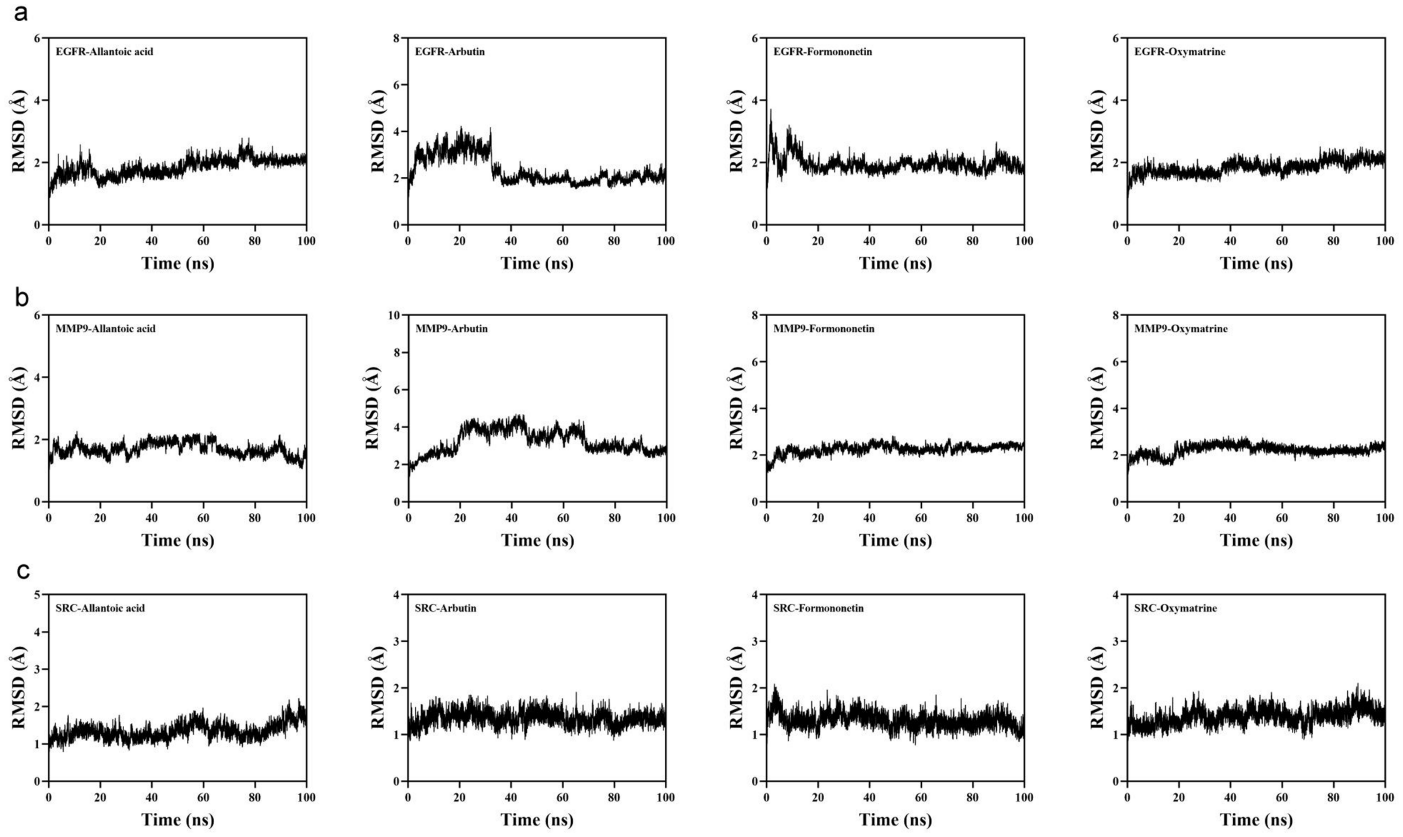
Visualization of molecular dynamics. **(a–c)** Root mean square deviation analysis.

To evaluate the dynamic behavior of allantoic acid, arbutin, formononetin, oxymatrine, and their binding proteins EGFR, MMP9, and SRC, molecular dynamics simulations were conducted. Root-mean-square deviation (RMSD) indicated that EGFR-allantoic acid, EGFR-arbutin, EGFR-formononetin, and EGFR-oxymatrine reached equilibrium after 80, 40, 20, and 40 ns, respectively, demonstrating stable and reliable simulations throughout the process (Figure 10a). Likewise, SRC-allantoic acid, SRC-arbutin, and SRC-formononetin exhibited overall stability (Figure 10b). MMP9-arbutin, MMP9-formononetin, and MMP9-oxymatrine also showed general stability (Figure 10c). Concurrently, radius of gyration (Rg) results indicated that EGFR-allantoic acid, EGFR-formononetin, EGFR-oxymatrine, MMP9-allantoic acid, SRC-allantoic acid, SRC-arbutin, and SRC-formononetin bind stably without inducing significant protein expansion or contraction (Supplementary Figure S4). In contrast, the EGFR-arbutin, MMP9-arbutin, MMP9-formononetin, and MMP9-oxymatrine complexes showed some changes, indicating conformational changes during the simulation. Subsequently, solvent-accessible surface area (SASA) analysis revealed that small molecules may influence the binding microenvironment upon protein interaction, leading to measurable SASA variations (Supplementary Figure S5).

**Figure 11:**
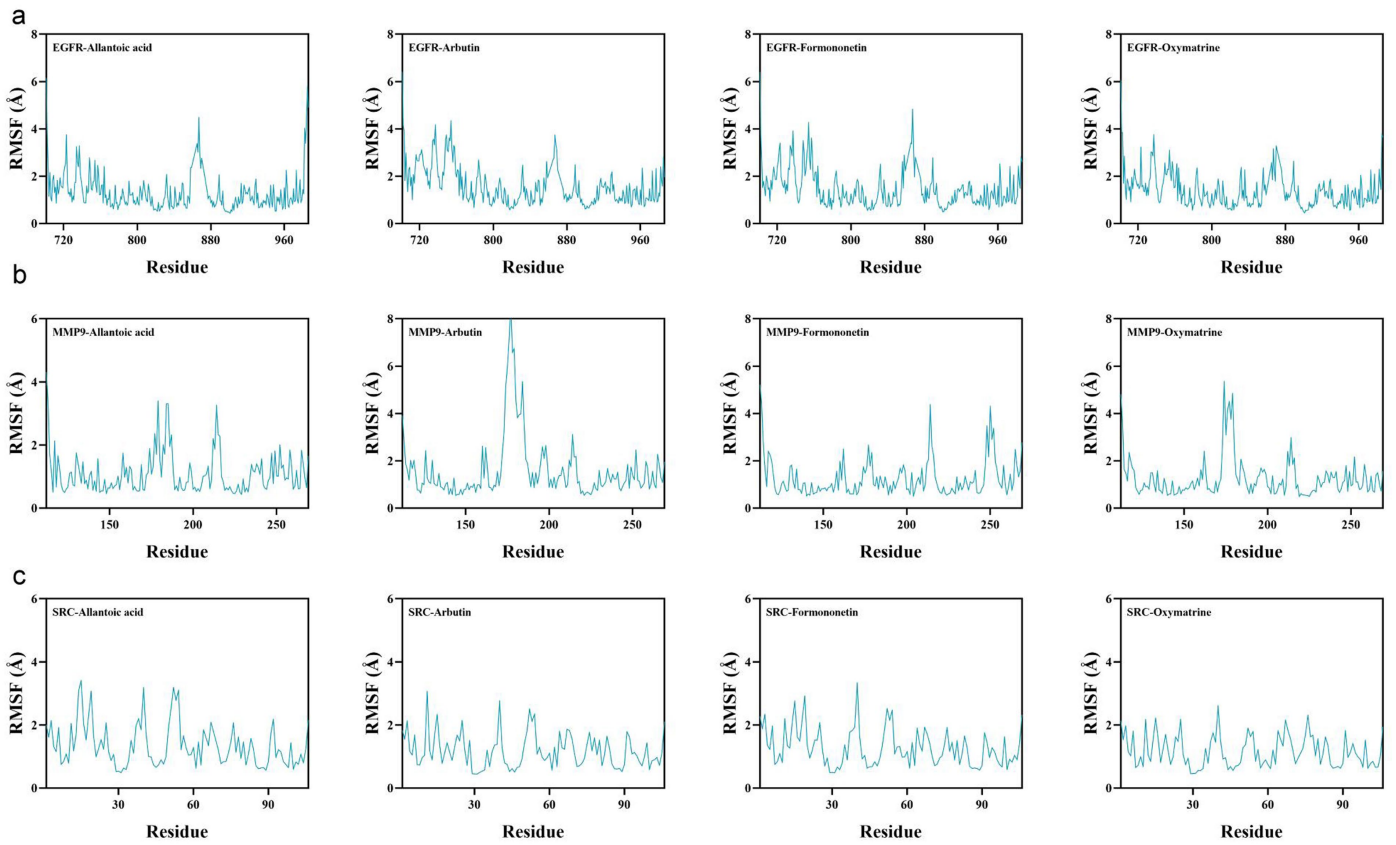
Visualization of molecular dynamics. **(a–c)** Root mean square fluctuation analysis.

Subsequently, root mean square fluctuation (RMSF) analysis characterized the flexibility of distinct protein regions. Results indicated that the small molecules exerted minimal structural influence on EGFR and SRC but showed greater effects on MMP9–oxymatrine and MMP9–arbutin (Figure 11). Taken together, these findings suggest that the proteins interact stably with the small molecules.

**Figure 12:**
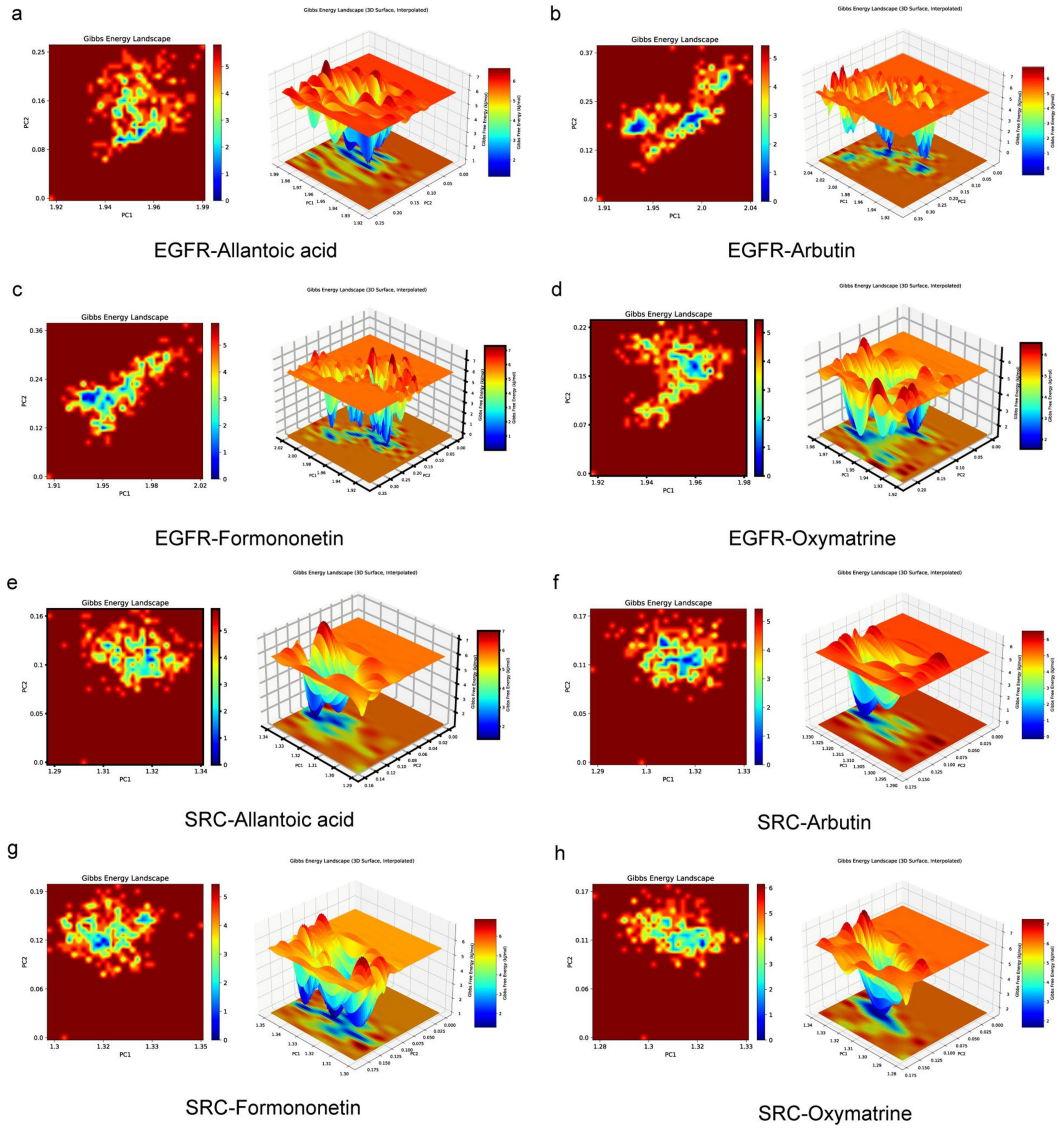
Visualization of free energy landscape. **(a)** EGFR-allantoic acid, **(b)** EGFR-arbutin, **(c)** EGFR-formononetin, **(d)** EGFR-oxymatrine, **(e)** SRC-allantoic acid, **(f)** SRC-arbutin, **(g)** SRC-formononetin, **(h)** SRC-oxymatrine.

Ultimately, to reflect intermolecular interactions and energy distribution within the binding system, free energy landscape plots were constructed. Results indicate that when EGFR binds to allantoic acid (Figures 12a–d), the low-energy region is located at a larger RMSD, exhibiting conformational features distinct from those of the EGFR-arbutin, EGFR-formononetin, and EGFR-oxymatrine complexes. The low-energy regions for MMP9 binding to arbutin, formononetin, oxymatrine, and allantoic acid exhibited similar distributions and favorable conformational stability (Supplementary Figure S6). In contrast, the low-energy regions for SRC binding to arbutin, formononetin, oxymatrine, and allantoic acid were more concentrated (Figures 12e–h), indicating high conformational stability. These findings suggest that allantoic acid, arbutin, formononetin, and oxymatrine in SF-PA-EVLPs may serve as potential components for treating diabetic wounds, with EGFR, MMP9, and SRC as key targets. These findings differ from previous studies, which primarily focused on a single ingredient to promote wound healing with a single target of action and efficacy, and could not fully alleviate symptoms (22). SF-PA-EVLPs, derived from the medicinal herb SF and insect PA, is an extracellular vesicle-like particle characterized by high safety and multicomponent, multitarget properties, offering potential for efficient treatment of diabetic wounds.

### 2.14 SF-PA-EVLPs influences diabetic wound healing by modulating the activity of EGFR, MMP9, and SRC

**Figure 13:**
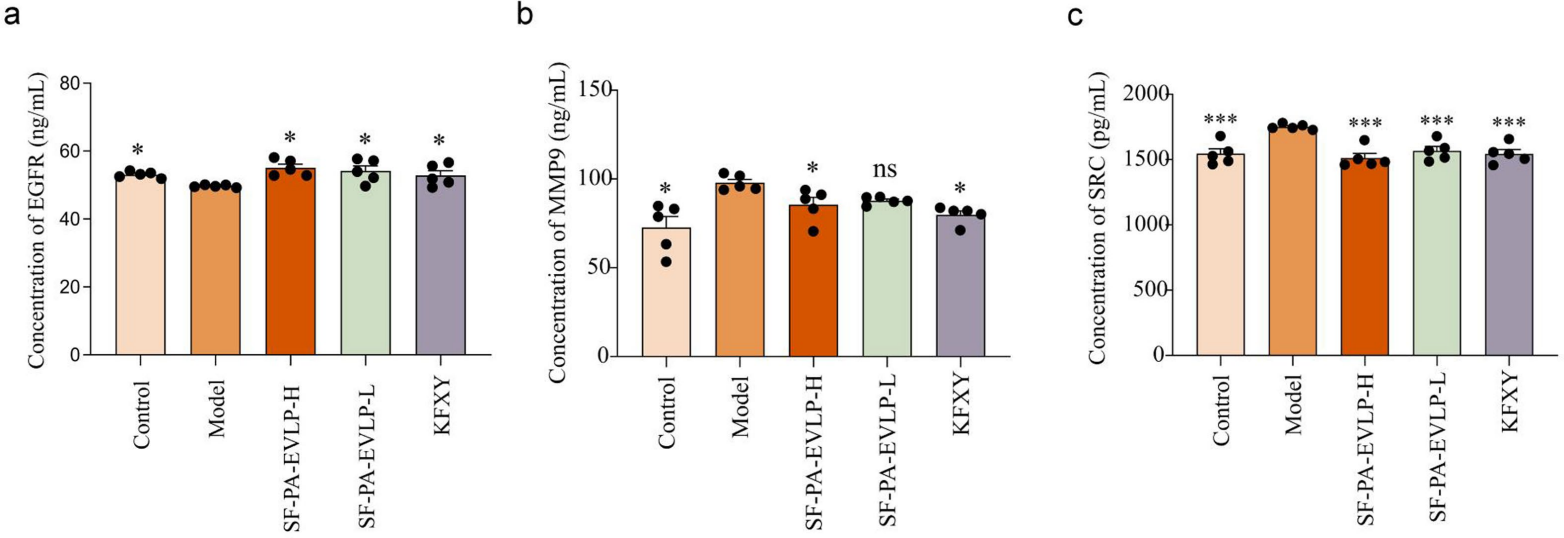
Measurement of relevant indicators in serum. **(a)** EGFR, **(b)** MMP9, **(c)** SRC.

EGFR is a well-known transmembrane glycoprotein and is highly associated with cell proliferation, migration, and differentiation (23). Concurrently, upregulation of EGFR expression promotes wound healing in diabetic wounds (24). Furthermore, the SRC protein is a non-receptor tyrosine kinase that can be activated by various signaling pathways, thereby triggering associated signaling cascades, including STAT and EGFR (25). Evidence indicates that inhibition of the SRC signaling pathway plays a therapeutic role in diabetic wound healing (26). Moreover, MMP9 is a matrix metalloproteinase highly expressed in diabetic wounds (27). Therefore, inhibition of MMP9 activity promotes the healing of diabetic wounds (28). To assess expression changes in the key signaling pathways identified through network pharmacology, ELISA assays were conducted to measure serum levels of EGFR, SRC, and MMP9. The results revealed that SF-PA-EVLPs significantly inhibited SRC and MMP9 expression while markedly promoting EGFR expression, thereby exerting a synergistic therapeutic effect on diabetic wounds (Figure 13). This further highlights the advantages of the multi-component, multi-target strategy of SF-PA-EVLPs in treating diabetic wounds.

### 2.15 Biocompatibility of SF-PA-EVLPs

To more effectively evaluate the biocompatibility of SF-PA-EVLPs, CCK8 assays were conducted. SF-PA-EVLPs was a combination of SF-EVLP (1 × 10^10^ particles/mL) and PA-EVLP (1 × 10^9^ particles/mL). The cell viability data indicated that this combination exhibited no significant cytotoxic effects on HUVECs (Supplementary Figure S7).

### 2.16 Cellular uptake of SF-PA-EVLPs

**Figure 14:**
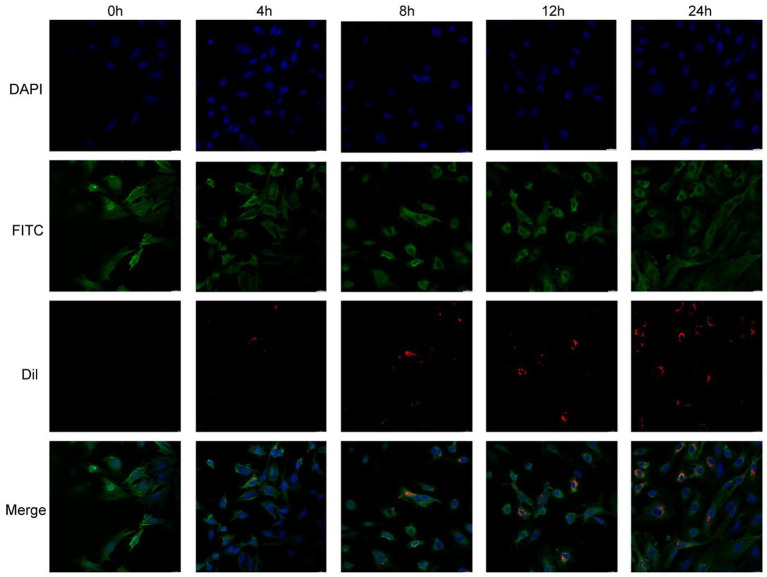
Representative images of the cellular uptake of SF-PA-EVLPs in HUVEC.

**Figure 15:**
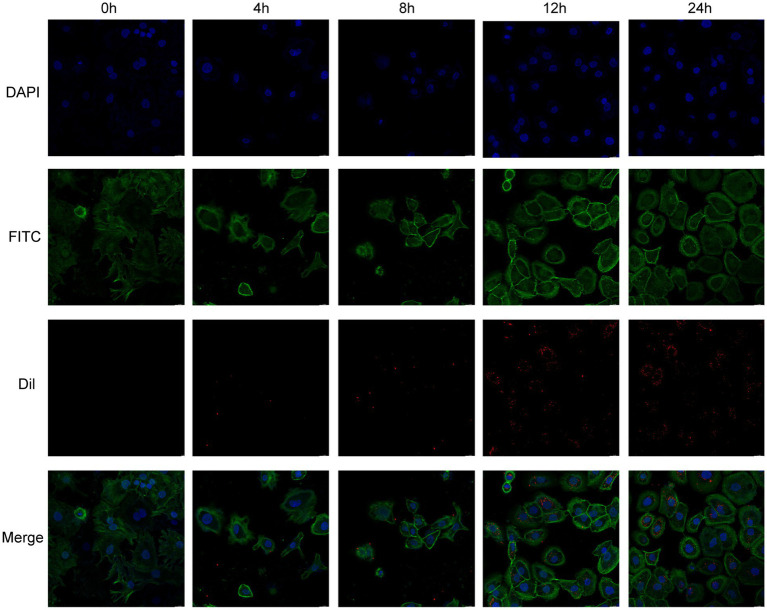
Representative images of the cellular uptake of SF-PA-EVLPs in HaCaT.

We observed that under our wide-field high-definition imaging system, DiI-labeled SF-PA-EVLPs were efficiently internalized by HUVEC and HaCaT cells (Figures 14, 15), with the uptake sites predominantly localized in the cytoplasm. As the co-culture time increased, the fluorescence signal carried by SF-PA-EVLPs gradually intensified, indicating time-dependent cellular uptake and progressive intracellular accumulation of SF-PA-EVLPs. This indicates that the nanoparticle size of SF-PA-EVLPs enables effective penetration into HUVEC and HaCaT cells, thereby enhancing intercellular transfer and bioavailability of insoluble bioactive substances.

### 2.17 SF-PA-EVLPs promotes wound healing in diabetes

**Figure 16:**
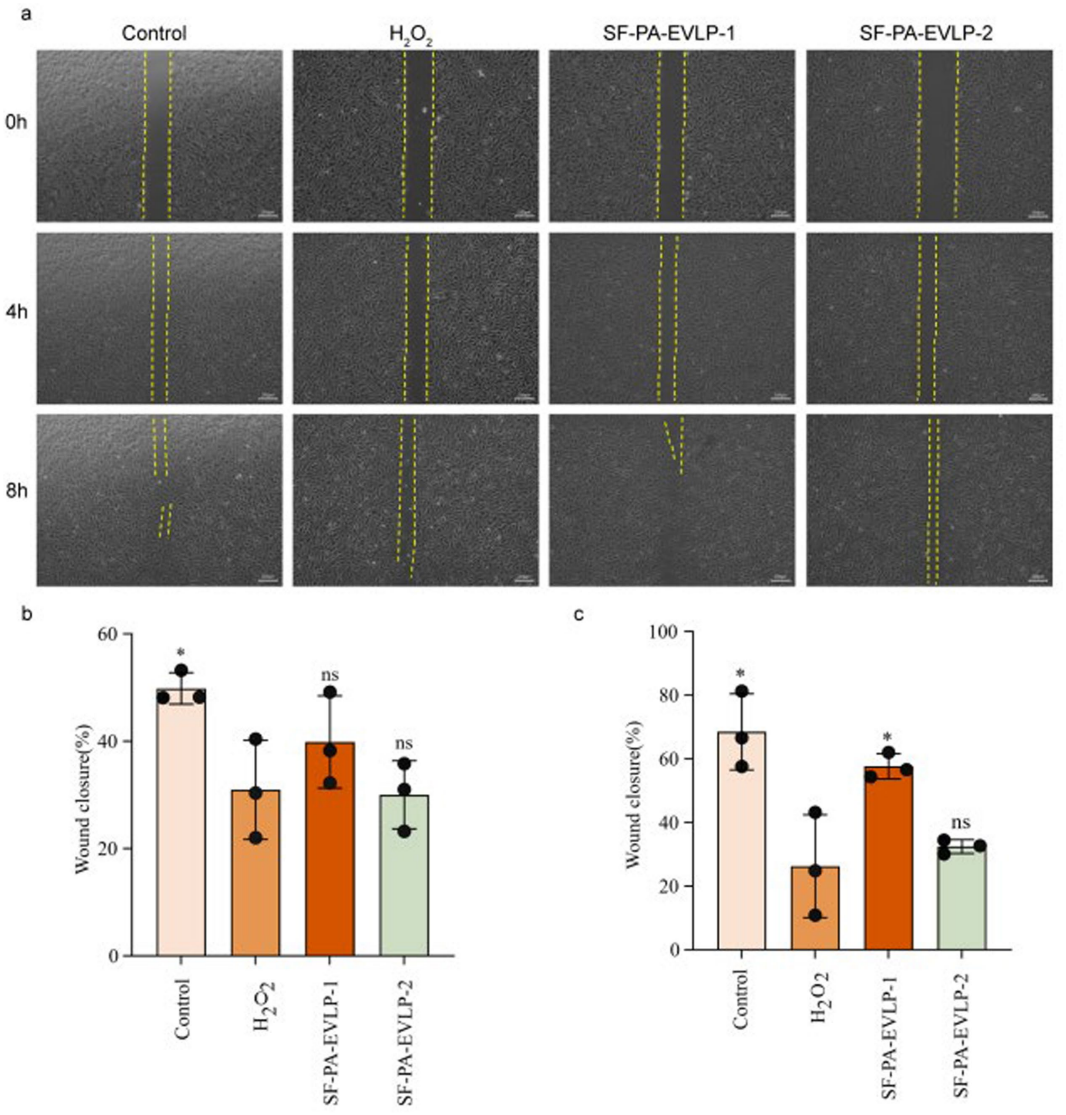
Scratch wound healing assay performed on HUVECs subjected to different treatments at 0, 4, and 8 h (*n* = 3). Scale bar, 200 μm. In vitro HUVECs wound healing assay. **(a)** Scratch wound healing assay performed on HUVECs subjected to different treatments at 0, 4, and 8 h (*n* = 3). Scale bar, 200 μm. **(b,c)** Quantitative analysis of wound closure rate at 4, and 8 h.

The cell migration capability of HUVECs under H_2_O_2_ stimulation was evaluated via a scratch assay. As shown in Figure 16, under high-glucose conditions, the addition of H_2_O_2_ markedly inhibited HUVEC migration, an effect that was reversed by SF-PA-EVLPs, suggesting that SF-PA-EVLPs has the potential to restore wound healing.

## 3 Discussion

Diabetic wounds aggravated by hyperglycemia and bacterial infection are a global challenge (29). Although PA extract is extensively used as a first-line therapeutic agent, it has significant limitations in this complex pathological environment (6). Thus, novel therapeutic strategies need to be developed urgently. Therapeutic approaches using EVLP have recently emerged as a promising new strategy in the comprehensive management of diabetic wounds. By simultaneously accelerating wound healing and inhibiting bacterial growth, EVLPs offer a dual-action approach to wound repair. The primary advantage of their application lies in their unique nanoscale phospholipid bilayer structure. While facilitating intercellular communication by delivering bioactive substances, they also increase the solubility, stability, and bioavailability of poorly soluble components through their small size (nanometer range) (11), enabling sustained-release effects (15, 30). This advanced concept aligns with the longstanding tradition of applying natural products for wound healing. Besides antibacterial medicinal plants such as SF (5, 31), insects and their extracts (e.g., PA) have also been used in wound management, as indicated by the formulation of Kangfuxin Liquid (6, 7). PA-EVLPs can accelerate diabetic wound healing (32). However, the therapeutic efficacy and the mechanisms underlying the action of a synergistic SF-PA-EVLPs system, formed by combining SF-EVLP with PA-EVLP, need to be investigated. Whether this combined system is better than the clinically used PA extract for diabetic wound treatment needs to be investigated. Therefore, systematic studies need to be conducted to address this research gap.

To evaluate whether SF-PA-EVLPs can effectively perform diabetic wound healing, we obtained high-quality SF-EVLP and PA-EVLP from SF and PA through a green extraction process, respectively. This is the first study to establish a plant-insect cross-species extracellular vesicle kingdom communication paradigm (SF-PA-EVLPs). The efficacy of SF-PA-EVLPs was subsequently compared to that of the clinically used PA extract. SF-EVLP, derived from the botanical source SF, has benefits such as natural origin, low toxicity, and high efficacy (21). PA-EVLPs, obtained from a medicinal insect, demonstrate high environmental adaptability and developmental plasticity.

This was the first study to use SF-EVLP and PA-EVLP as advanced natural bioactive entities. This strategy shifts away from conventional paradigms of natural product extraction and has multiple innovative implications. Both SF-EVLP and PA-EVLP have a nanoscale lipid bilayer vesicle structure, which imparts them distinct “natural nanocarrier” properties. In contrast to traditional ethanol extracts, EVLP can effectively encapsulate a comprehensive array of bioactive components from their nutritional content, including lipids, proteins, nucleic acids, and secondary metabolites, thereby forming structured functional complexes. In this study, SF-EVLP concentrated characteristic alkaloids such as matrine, while PA-EVLP retained distinctive constituents of PA, including proteins, amino acids, and arbutin. These components are preserved in a vesicle-encapsulated form, preventing denaturation and inactivation during processing while substantially improving bioavailability. Furthermore, SF-PA-EVLPs exhibits excellent uptake by extracellular HUVECs and HaCaT. This may be attributed to the nanoparticle size and lipid bilayer membrane structure of RD-EVLP, which enhances its affinity for cells and facilitates intercellular communication between plant vesicles and animal cell membranes.

The combination of SF-EVLP and PA-EVLP constitutes a functionally complementary therapeutic system, achieving a synergistic effect in the treatment of diabetic wounds. This plant-insect cross-kingdom combination leverages the evolutionary divergence between the two types of EVLPs: plant-derived EVLPs are rich in polyphenols and flavonoids with antioxidant properties, whereas insect-derived EVLPs contain unique components such as proteins, amino acids, and arbutin. This combination thereby achieves a molecular diversity that surpasses what any single source can provide. The lipid bilayer structure of both SF-EVLP and PA-EVLP provides a physical protective barrier for the encapsulated active ingredients. The wound healing rate in the combined EVLP treatment group was significantly greater than that in the PA extract group, suggesting that the vesicular structure effectively delays the degradation of the components of PA. This structure–function coupling characteristic represents the core competitive advantage of EVLPs as a novel bioactive substance.

Besides promoting local wound healing, we observed that pancreatic islet damage was less severe in the SF-PA-EVLPs group compared to the model group. Nevertheless, additional evidence is needed to establish that SF-PA-EVLPs are effective in ameliorating pancreatic injury in diabetic rats. Finally, through an integrated approach involving network pharmacology, molecular docking, molecular dynamics, and ELISA, we revealed that SF-PA-EVLPs may treat diabetic wounds by modulating key signaling pathways such as EGFR, highlighting the multi-component synergistic mechanism underlying their therapeutic action. To summarize, our investigation into the plant-insect-mammal cross-kingdom EVLP composite system offers a promising new paradigm for diabetic wound management. We propose a natural, efficient, and safe therapeutic strategy that incorporates the translational application of bioactive compounds from plants and insects into pharmaceuticals, while also providing an excellent model for interdisciplinary innovation across food science, nutrition, and pharmaceutical research. Moreover, both SF-EVLP and PA-EVLP exhibit excellent biocompatibility and safety profiles. Plant-derived EVLPs have lower immunogenicity than animal-derived EVLPs, and the combination of EVLPs from plants and insects in this study did not lead to additive immunogenicity or synergistic toxicity. This favorable safety profile provides an important foundation for clinical translation, particularly in the long-term management of chronic diabetic wounds.

Several challenges still exist with respect to the cross-kingdom regulatory effects of plant-derived vesicles on mammals. It has been found that repeated administration of exogenous EVLPs may trigger immune memory and the production of specific antibodies, leading to faster clearance from the body and reduced effectiveness over time. In terms of clinical application, several practical challenges remain, including low production yields, inefficient isolation methods, and notable difficulties in ensuring the purity of PD-EVLPs. While current guidelines often lack detailed assessment of immune-related profiles, available preclinical studies suggest negligible toxicity (33). Moreover, controlled plant-based production systems for scalable, high-purity, and low-cost preparation of these EVLPs are still in the early stages of development and require substantial clinical validation. This study conducted a preliminary dermal safety evaluation via *in vitro* and *in vivo* experiments, and no significant systemic toxicity or tissue damage was observed. However, OECD 402 acute irritation and corrosion tests are lacking, which is a limitation. Future studies should include these standardized tests to refine dermal safety assessment and promote clinical translation.

## 4 Conclusion

This was the first study to construct and validate the plant-insect extracellular vesicle cross-kingdom paradigm (SF-PA-EVLPs); our results showed that these SF-PA-EVLPs have remarkable efficacy in diabetic wound healing. This is attributed to the cross-species bioactivity integration capability of SF-PA-EVLPs and their multi-component synergistic regulation of the EGFR signaling pathway to accelerate wound healing. The SF-PA-EVLPs therapeutic system continuously promotes wound healing through a multi-target synergistic mechanism while demonstrating excellent safety throughout the treatment period. As an innovative and sustainable strategy based on the “plant-insect-mammal” cross-kingdom communication paradigm, this study offered a safe and effective potential alternative for treating chronic wounds and related conditions. It also provided a scientific rationale and technical pathways for the comprehensive development of medicinal plants, insects, and dual-purpose nutritional-food resources, as well as their transformation into functional foods.

## 5 Materials and methods

### 5.1 Extraction and identification of SF-EVLP and PA-EVLP

*Sophora flavescens*-derived extracellular vesicle-like particles (SF-EVLP) and *Periplaneta americana-d*erived extracellular vesicle-like particles (PA-EVLP) were extracted following the protocol described in another study (9). Briefly, 200 g of herb and insects of RD were squeezed, and the obtained juice was filtered. Larger particles were removed via differential centrifugation at sequential speeds of 500 × *g*, 2,000 × *g*, 5,000 × *g*, and 10,000 × *g* for several minutes. Then the pellet was extracted from the centrifugation tube. The supernatant underwent centrifugation, and the pellet was resuspended in PBS and filtered through a 0.22 μm membrane. The samples were kept at 4 °C for less than 7 days or frozen at −80 °C. Freeze–thaw cycles were not performed more than once.

### 5.2 NanoFCM detection of SF-EVLP and the diameter and concentration of PA-EVLP

The diameter and concentration of SF-EVLP and PA-EVLP were determined by NanoFCM, a technique for determining the size distribution profiles of small particles in suspensions using known standards. The samples were diluted 1:1,000 and analyzed using a Flow Nano Analyzer (Xiamen Fuliu Biotechnology Co., Fujian, China) following the manufacturer’s instructions. A 250 nm quality control particle calibration laser served as the reference for the concentration of particles. A particle mixture ranging from 68 to 155 nm was used to establish the reference diameter distribution curve (9).

### 5.3 Transmission electron microscopy detection of SF-EVLP and PA-EVLP

A sample solution (5–10 μL) was added dropwise onto a copper grid. After rinsing with PBS, the grid was negatively stained with phosphotungstic acid, air-dried for 5 min, and examined using a JEOL-400 TEM (Japan) at 80–120 kV.

### 5.4 Purity assessment of SF-EVLP and PA-EVLP

The Triton X-100 membrane rupture test indirectly indicates the purity of SF-EVLP and PA-EVLP. The SF-EVLP and PA-EVLP were equally divided into two test tubes each. Triton X-100 was added to each tube at a final concentration of 0.1% (v/v in PBS), followed by vigorous mixing for 30 s. The mixture was incubated for 30 min at room temperature and the number of particles of SF-EVLP and PA-EVLP were determined using a Flow NanoAnalyzer.

### 5.5 Metabolomics analysis

The phytochemicals in SF-EVLP were qualitatively analyzed using ultra-high-performance liquid chromatography coupled with mass spectrometry (UHPLC/MS). The samples were analyzed using a 2.1 × 100 mm Waters ACQUITY UPLC HSS T3 column with a particle size of 1.8 μm. In both the positive and negative electrospray ionization (ESI) modes, the mobile phase included two components: 0.1% formic acid in water as component A and 0.1% formic acid in acetonitrile as component B. The gradient elution conditions were as follows: 0–5 min transitioning from 5 to 65% B, 5–6 min transitioning from 65 to 99% B, 6–7.5 min at 99% B, and 7.5–10 min transitioning from 5% B. The flow rate measured was 0.4 mL/min. The ESI source conditions were configured with a sheath gas setting of 30 Arb and an auxiliary gas setting of 5 Arb. In MS1-only acquisition, the data were recorded over an *m*/*z* range of 84–1,250 Da with a resolution of 35,000.

The phytochemicals in PA-EVLP were qualitatively analyzed using UHPLC/MS. The samples were analyzed using a 2.1 × 100 mm Waters ACQUITY Premier HSS T3 column with 1.8 μm particle size. In both positive and negative ESI modes, the mobile phase includes two components: 0.1% formic acid in water as component A and 0.1% formic acid in acetonitrile as component B. The gradient elution conditions were as follows: 0–2 min transitioning from 5 to 20% B, 2–5 min transitioning from 20 to 60% B, 5–7.5 min transitioning from 60 to 99% B, and 7.5–10 min transitioning from 99 to 5% B. The flow rate measured was 0.4 mL min^−1^. The ESI source conditions were configured with a sheath gas setting of 60 Arb and an auxiliary gas setting of 20 Arb. In MS1-only acquisition, the data were recorded over an *m*/*z* range of 70–1,000 Da with a resolution of 60,000.

### 5.6 Animal experiment

Male Sprague–Dawley rats (*n* = 25; weight: 150 ± 20 g) were obtained from Guangzhou Ruiye Model Animal Center, and the study was approved by the Laboratory Animal Ethics Committee of Guangzhou Ruiye Model Animal Biotechnology Co. (Protocol No. RYEth-202509301080). All animal experiments followed the Animal Research: Reporting of *In Vivo* Experiments (ARRIVE) guidelines. The rats were housed under specific pathogen-free laboratory conditions. After one week of adaptive feeding, STZ (50 mg kg^−1^) was injected intraperitoneally. Then, the rats were observed every day. After 3 days, blood was collected from the tail vein to measure the random blood glucose level; rats with random blood glucose levels >16.7 mmol L^−1^ were considered to be diabetic. For the diabetic full-thickness back skin wound model, diabetic rats were randomly divided into four groups: model, SF-PA-EVLP with SF-EVLP 1 × 10^10^ particles mL^−1^and PA-EVLP 1 × 10^9^ particles mL^−1^ (low concentration), SF-PA-EVLP (high concentration) with SF-EVLP 1 × 10^11^ particles mL^−1^and PA-EVLP 1 × 10^10^ particles mL^−1^, and KFXY. Full-thickness wounds were made on the back skin using a 10 mm diameter punch biopsy device. No drug was administered to the rats in the control group. The wound healing process was photographed by a digital camera, and the size of the wound was quantified by the software ImageJ. At the end of the experimental period, the rats were fasted at the beginning of the active phase for 12 h. Subsequently, the animals were deeply anesthetized using 5% isoflurane (Hebei Jindafu Pharmaceutical Co., Ltd., China) inhalation with a gas anesthesia apparatus (Rewode, Shenzhen, China). Once deep anesthesia was confirmed (loss of righting reflex and pain response), whole blood samples were collected via abdominal aorta until death. After confirming cessation of breathing and heartbeat and pupillary dilation, isoflurane administration was discontinued. Subsequently, tissues including the heart, liver, spleen, lungs, kidneys, pancreas, femurs, and tibiae were collected from each group of rats.

### 5.7 Biocompatibility of SF-PA-EVLPs

The serum levels of alanine aminotransferase (ALT), aspartate aminotransferase (AST), alkaline phosphatase (ALP), blood urea nitrogen (BUN), uric acid (UA), and blood creatinine (CREA) were quantified using an automated biochemical analyzer (Beckman AU5800 Chemistry Analyzer, Beckman Coulter, United States).

### 5.8 H&E staining

Wound tissues, heart, liver, spleen, lungs, kidneys, and pancreas were collected after the animals were euthanized on day 14. Subsequently, the tissues were preserved in 4% paraformaldehyde overnight, embedded in paraffin, and sliced into thin sections (approximately 5 μm thick) for H&E staining using H&E staining kits (G1076, G1101; Servicebio, China). The histology images were captured under a microscope.

### 5.9 Masson staining

Wound tissues were collected after the animals were euthanized on day 14. These tissues were preserved in 4% paraformaldehyde overnight, embedded in paraffin, and sliced into thin sections (approximately 5 μm thick) for Masson staining. The histology images were captured under a microscope and quantified using NDP.view2 and ImageJ software. Subsequently, histological assessment involved fixing the knees in 10% neutral formalin for 48 h, followed by decalcification in 10% EDTA (pH 7.4) at 37 °C and 80 rpm for 5 days. Following gradient dehydration, the knees were embedded in paraffin and sectioned sagittally at 4 μm for histological analysis using Masson staining kits (G1006, G103; Servicebio, China).

### 5.10 ELISA

An appropriate volume of serum was added to the sample wells (Meimian, Jiangsu, China). Parallel blank wells and wells containing serial dilutions of the standard were also prepared. A 100 μL volume of biotinylated antibody working solution was dispensed into each well, followed by resealing and incubation at 37 °C for 60 min. After incubation, the solution was aspirated and the membrane was allowed to dry. The wells were then washed by adding 300 μL of wash solution to each well, and the wells were soaked for 1–2 min, and the blot was dried; this step was repeated 5 times. Next, 50 μL of substrate A solution and 50 μL of substrate B solution were added to each well, followed by sealing the plate and incubating it at 37 °C for 15 min in the dark. The reaction was terminated by the addition of 50 μL of stop solution, followed by incubation at room temperature until the color transitioned completely from blue to yellow. The OD_450_ was measured within 15 min using an ELISA microplate reader.

### 5.11 Network pharmacology analysis

First, the potential targets for SF-EVLP and FS-EVLP components were identified and collected using the Swiss-PTA database.^1^ Subsequently, diabetic wound healing targets were identified using multiple databases, including GeneCards^2^ and OMIM.^3^ Targets collected from these databases were consolidated, and duplicates were removed. Next, we performed the following analyses: acquisition of intersecting genes associated with the use of SF-PA-EVLPs for diabetic wound healing, construction of a protein–protein interaction network, establishment of an SF-PA-EVLP–component–target–disease network, screening of key targets, protein clustering analysis, and functional enrichment analysis, including GO and KEGG pathway analysis.

### 5.12 Molecular docking

First, the 2D structure of the small-molecule ligand was obtained from the PubChem database and converted into a 3D structure using the Chem Office software, which was then saved in the mol2 format. Simultaneously, a high-resolution protein crystal structure was selected from the RCSB PDB database. Water molecules and heteroatoms, such as phosphate groups, were removed using PyMOL, and the resulting structure was saved as a PDB file. Subsequently, AutoDock was used to add hydrogen atoms, calculate charges, determine torsional flexibility, and define the docking box coordinates for both the protein and ligand. Next, molecular docking was performed using AutoDock Vina, with the optimal conformation selected based on binding energy scores. Finally, PyMOL and Discovery Studio were used to generate 2D interaction diagrams and perform 3D visualization analysis to elucidate the binding mode between the compound and key residues.

### 5.13 Molecular dynamics simulation

Molecular dynamics simulations were conducted using GROMACS 2022. Protein force field parameters were generated with pdb2gmx, while ligand parameters were prepared using Sobtop based on the GAFF2 force field. The system was solvated and neutralized with 0.15 M NaCl using the gmx genion tool. Energy minimization was performed without constraints. Long-range electrostatic interactions were treated using the particle mesh Ewald (PME) method with a 1 nm cutoff. Bond constraints were applied using the LINCS algorithm. The system was maintained at 310 K using a Nosé–Hoover thermostat and at 1 bar using a Parrinello–Rahman barostat.

### 5.14 Cytocompatibility investigation

Primary chondrocytes were seeded in 96-well plates at 8,000 cells per well and incubated overnight (37 °C, 5% CO_2_). SF-PA-EVLP with varying particle concentrations (SF-EVLP 1 × 10^10^ particles/mL + PA-EVLP 1 × 10^9^ particles/mL; SF-EVLP 5 × 10^9^ particles/mL + PA-EVLP 5 × 10^8^ particles/mL; SF-EVLP 2.5 × 10^9^ particles/mL + PA-EVLP 2.5 × 10^8^ particles/mL; SF-EVLP 1 × 10^9^ particles/mL + PA-EVLP 1 × 10^8^ particles/mL) was then added. After 24 h, the medium was replaced with 100 μL of fresh medium containing 10 μL CCK-8 reagent, followed by incubation for 1.5 h at 37 °C. Absorbance was measured at 450 nm using a microplate reader (Rayto RT-2100C).

### 5.15 Scratch assays

HUVECs were seeded in 6-well plates until 90% confluence. Scratches were made in the 6-well plates using a 200 μL pipette tip. The corresponding drug treatments were administered, and the scratches were observed at 0 h, 4 h and 8 h using a light microscope and analyzed using ImageJ.

### 5.16 Cellular uptake of SF-PA-EVLPs

The uptake of SF-PA-EVLPs by cells was evaluated via laser confocal imaging (STELLARIS, Leica). After 24 h of exposure to SF-PA-EVLPs solution labeled with DiI, 0.1% Triton was applied for permeabilization. The cells were then washed again with PBS and incubated with FITC (CA1620, Solarbio, China) at room temperature in the dark for 20 min. After the slides were mounted with DAPI solution (C0060, Solarbio, China), fluorescence imaging was performed using a confocal laser scanning microscope.

### 5.17 Statistical analysis

All data were presented as the mean ± standard deviation from at least three independent experiments. The normality of the sample distribution was assessed by conducting the Shapiro–Wilk normality test. The statistical significance of all comparisons among and between groups was assessed using GraphPad Prism (version 8.0). To determine the differences among multiple groups, a one-way analysis of variance with Tukey’s *post-hoc* test was conducted. All differences were considered to be statistically significant at *p* < 0.05.

## Data Availability

The original contributions presented in the study are included in the article/Supplementary material, further inquiries can be directed to the corresponding authors.

## Footnotes

1
http://swisstargetprediction.ch/

2
https://www.genecards.org/

3
https://omim.org/
