# Correction: Nutritional properties of extracellular vesicle-like particles from *Sophora flavescens* and *Periplaneta americana* with effects on streptozotocin-induced diabetic wound healing in rats

**DOI:** 10.3389/fnut.2026.1881249

**Published:** 2026-05-28

**Authors:** Zhengting Wu, Yuanxin Zhao, Weiyin Zeng, Zesheng Lu, Qi You, Yingqi Cao, Yuanyuan Xia, Wencong Shen, Zilin Ou, Xiuping Cai, Qing Zhao, Kewei Zhao

**Affiliations:** 1 The Third Clinical Medical College, Guangzhou University of Chinese Medicine, Guangzhou, China; 2 State Key Laboratory of Traditional Chinese Medicine Syndrome, The Third Affiliated Hospital of Guangzhou University of Chinese Medicine, Guangzhou, China; 3 Guangdong Engineering Research Center of Chinese herbal-derived vesicles, Guangzhou University of Chinese Medicine, Guangzhou, China; 4 Guangdong Pilot Platform for Chinese Herbal Medicine-derived Extracellular Vesicles-like Particles, The Third Affiliated Hospital of Guangzhou University of Chinese Medicine, Guangzhou, China

**Keywords:** cross-kingdom modulation, diabetic wound, EGFR signaling pathway, *Periplaneta americana*-derived extracellular vesicle-like particles, *Sophora flavescens*-derived extracellular vesicle-like particles

In the published article, Figure 14 is a duplicate of Figure 16. The corrected Figure 14 and its caption appear below

**Figure 14:**
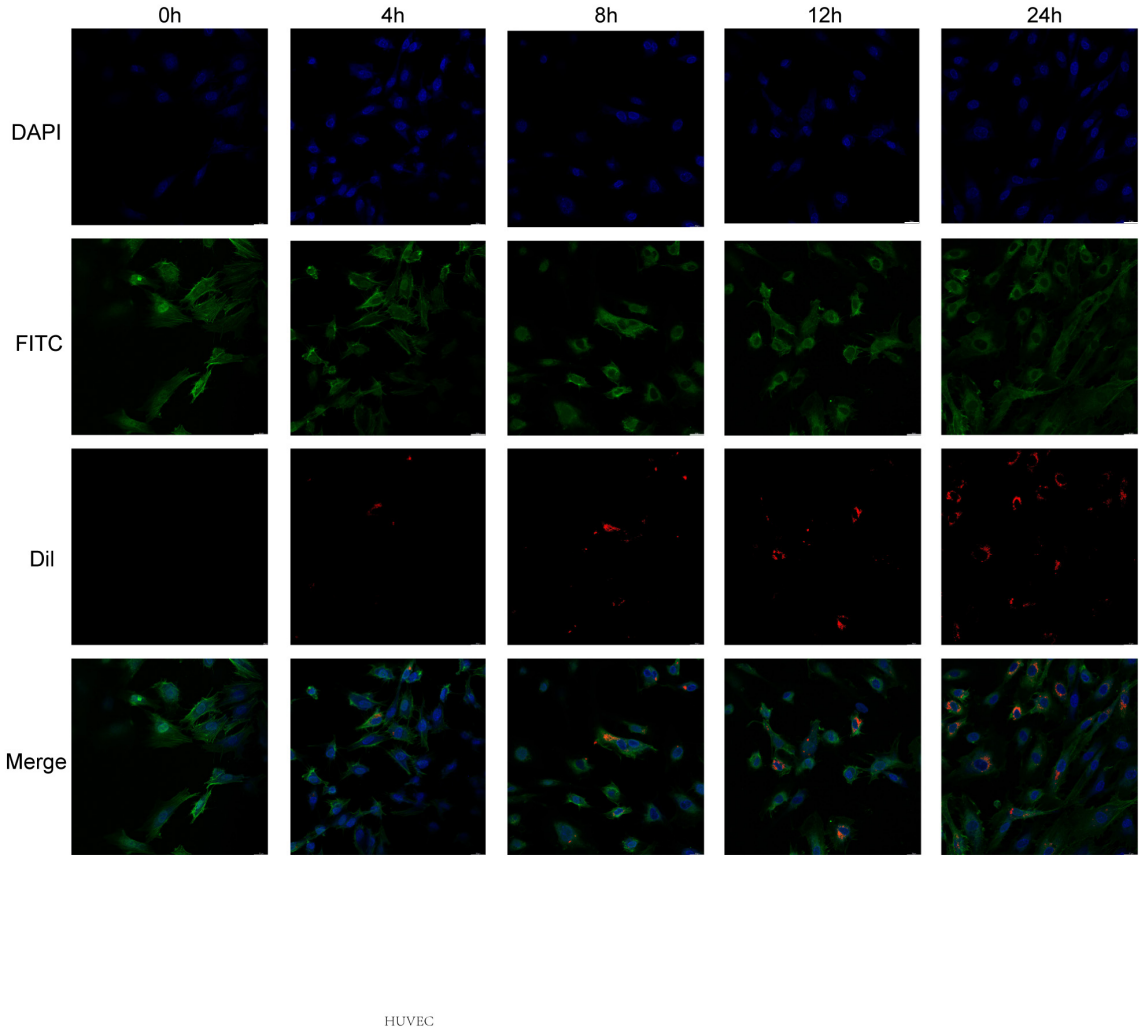
Representative images of the cellular uptake of SF-PA-EVLPs in HUVEC.

The original version of this article has been updated.

