# Supplementary material for: Nutritional properties of extracellular vesicle-like particles from Sophora flavescens and Periplaneta americana with effects on streptozotocin-induced diabetic wound healing in rats
Source: Front Nutr. 2026 Apr 29;13:1807919. doi: 10.3389/fnut.2026.1807919 (PMC13168163; doi:10.3389/fnut.2026.1807919)
Supplement: Supplementary file 1 [file Supplementary_file_1.docx]

**Supplementary Information**

Nutritional properties of extracellular vesicle-like particles from *Sophora flavescens* and *Periplaneta americana* with effects on streptozotocin-induced diabetic wound healing in rats

**This file includes:**

Figure S1 to S7.


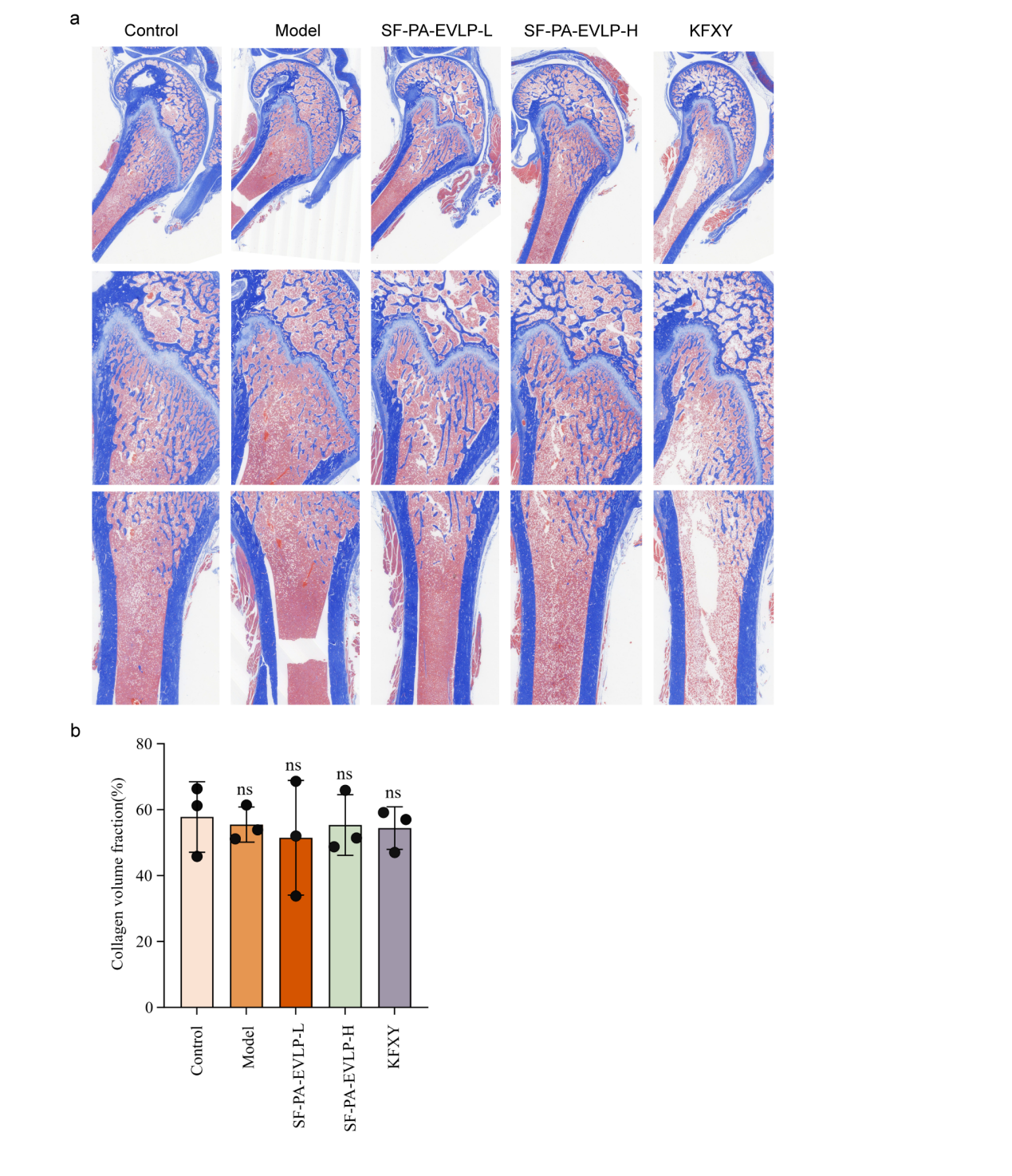


**Figure S1**. Representative images from Masson's trichrome staining of rat femurs are displayed. a) Masson staining of the femur; b) Collagen volume fraction.


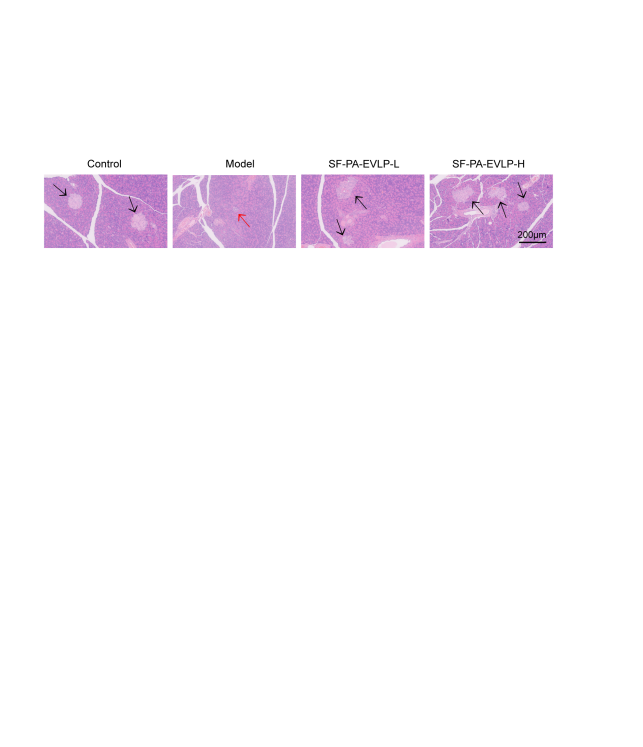


**Figure S2**. Effect of PS-EVLP on pancreatic histopathology in STZ-induced diabetic rats (n = 3; scale bar: 200 μm).


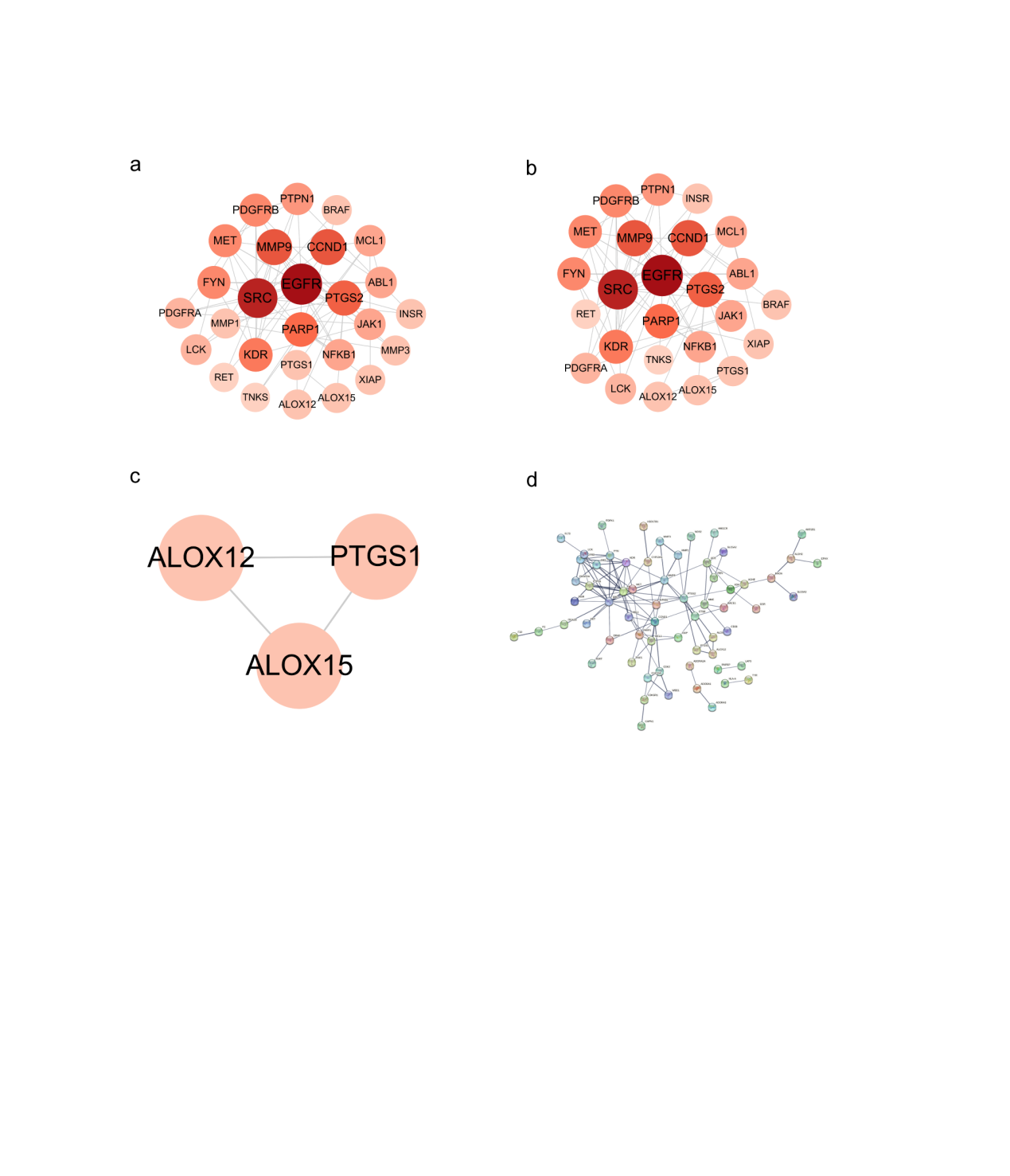


**Figure S3.** Network Pharmacology Analysis of SF-PA-EVLPs and diabetic wounds. a-c) Protein Cluster Analysis,Cluster1-3. d) Protein-protein interaction network.


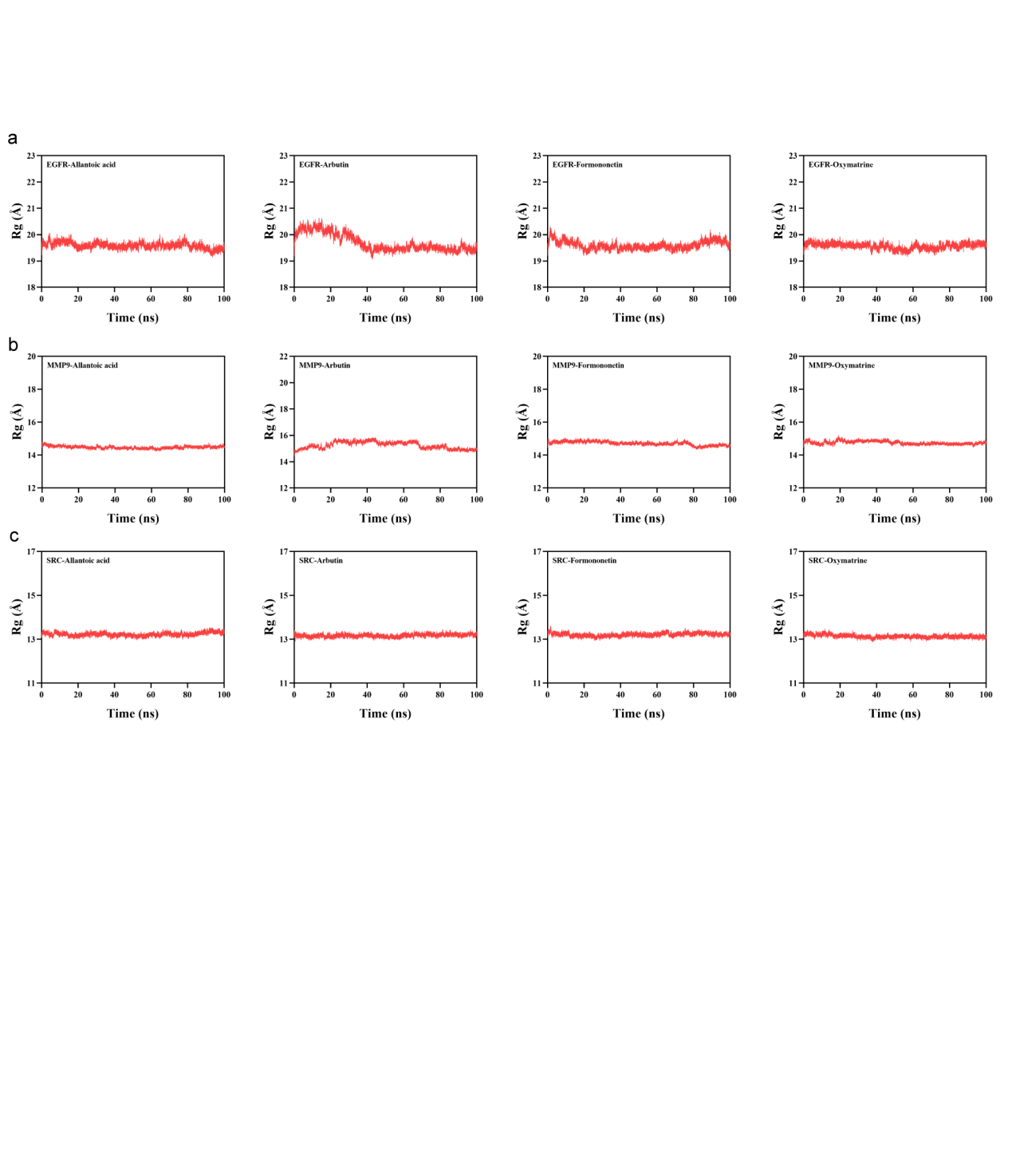


**Figure S4.** Visualization of molecular dynamics. a-c) Radius of gyration analysis.


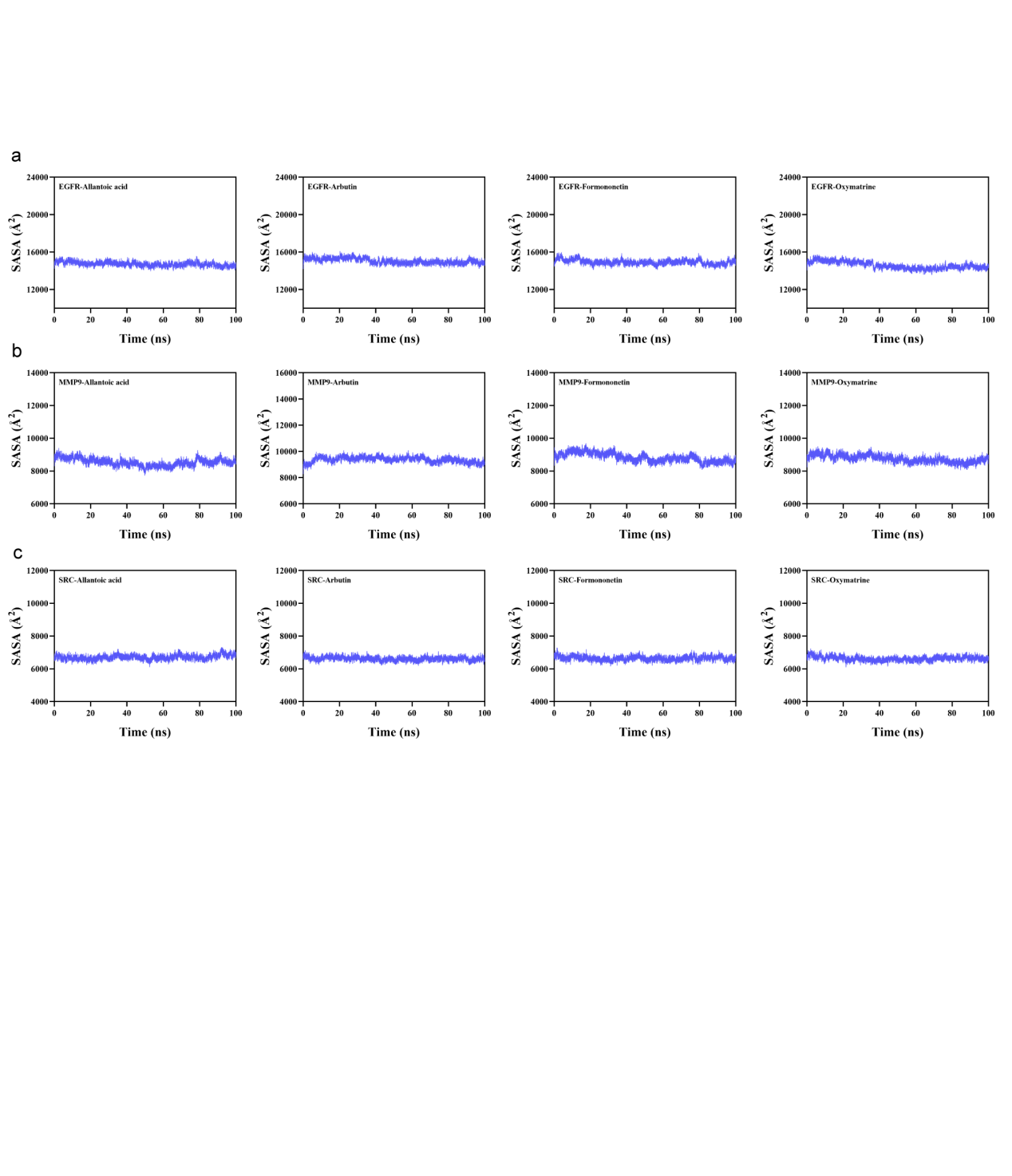


**Figure S5.** Visualization of molecular dynamics. a-c) Radius of gyration and the solvent-accessible surface areas analysis.


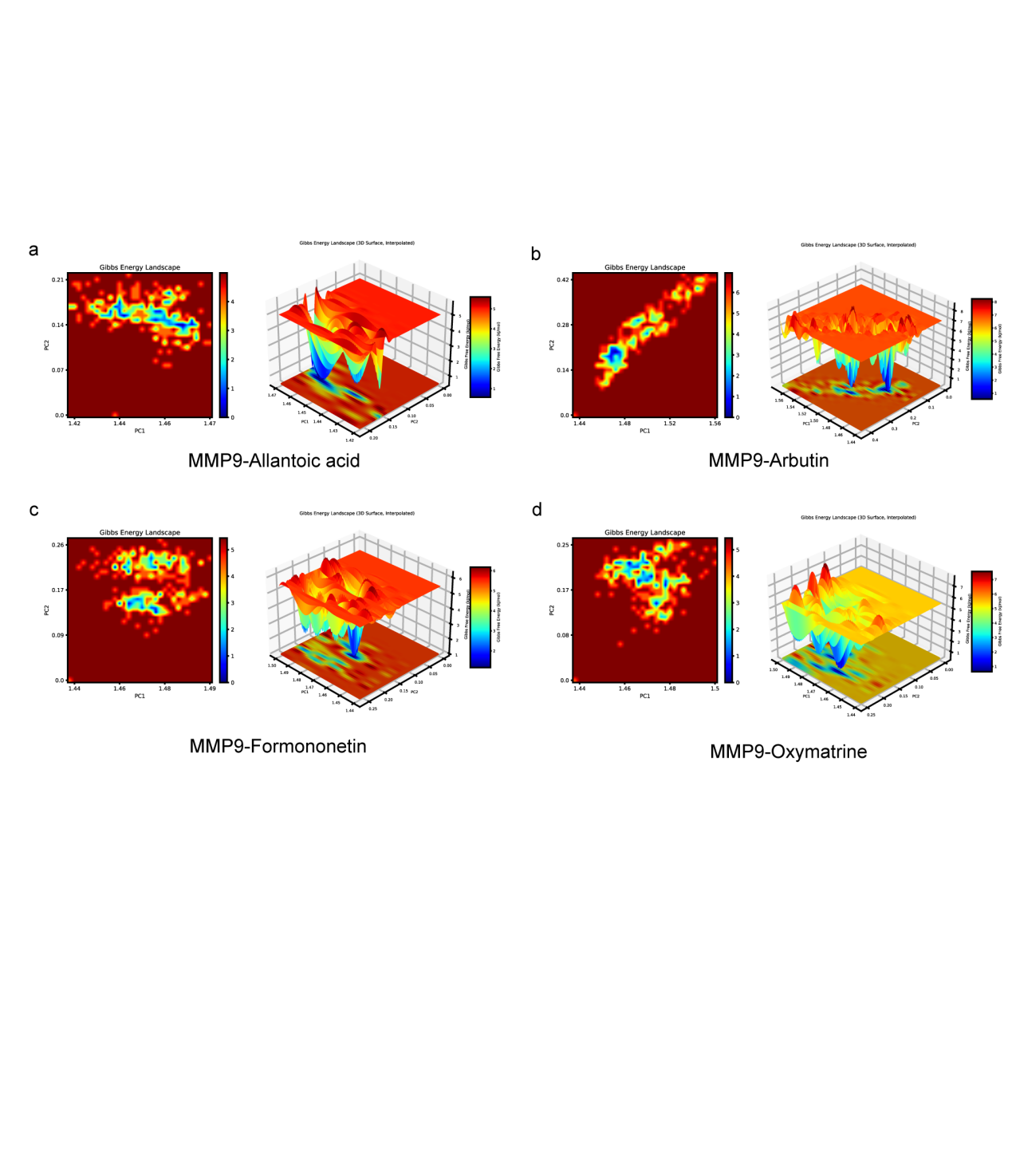


**Figure S6.** Visualization of free energy landscape of MMP9-conponments. (a) MMP9-Allantoic acid, (b) MMP9-Arbutin, (c) MMP9-Formononetin, (d) MMP9-Oxymatrine.


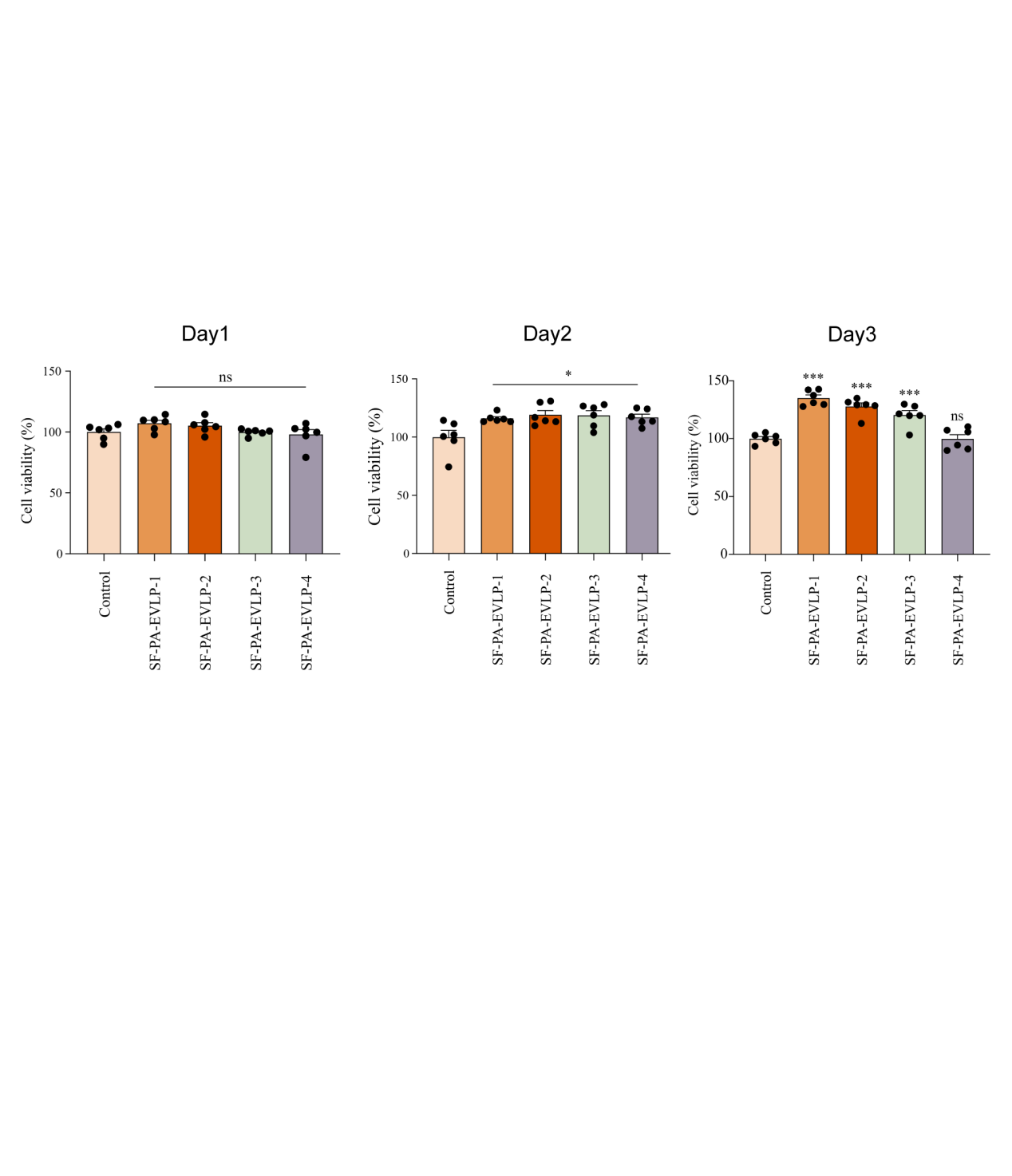


**Figure S7.** The viability of HUVECs cocultured with SF-PA-EVLP was detected by conducting a Cell Counting Kit-8(CCK8) assay for 1, 2, and 3 days (n = 6)
